# A rabies virus-vectored vaccine expressing two copies of the Marburg virus glycoprotein gene induced neutralizing antibodies against Marburg virus in humanized mice

**DOI:** 10.1080/22221751.2022.2149351

**Published:** 2022-12-28

**Authors:** Jinhao Bi, Haojie Wang, Qiuxue Han, Hongyan Pei, Hualei Wang, Hongli Jin, Song Jin, Hang Chi, Songtao Yang, Yongkun Zhao, Feihu Yan, Liangpeng Ge, Xianzhu Xia

**Affiliations:** a College of Veterinary Medicine, Jilin Agricultural University, Changchun, People’s Republic of China; b Changchun Veterinary Research Institute, Chinese Academy of Agricultural Sciences, Changchun, People’s Republic of China; c Institute of Laboratory Animal Science, Chinese Academy of Medical Sciences and Comparative Medicine Center, Peking Union Medical College (PUMC), Beijing, People’s Republic of China; d College of Chinese Medicinal Materials, Jilin Agricultural University, Changchun, People’s Republic of China; e Key Laboratory of Zoonosis Research, Ministry of Education, College of Veterinary Medicine, Jilin University, Changchun, People’s Republic of China; f Ruminant Disease Research Center, College of Life Sciences, Shandong Normal University, Jinan, People’s Republic of China; g Chongqing Academy of Animal Sciences, Chongqing, People’s Republic of China

**Keywords:** Marburg virus disease, Marburg virus, neutralizing antibodies, fully humanized antibody, transgenic mice, CAMouse, MARV vaccine

## Abstract

Marburg virus disease (MVD) is a lethal viral haemorrhagic fever caused by Marburg virus (MARV) with a case fatality rate as high as 88%. There is currently no vaccine or antiviral therapy approved for MVD. Due to high variation among MARV isolates, vaccines developed against one strain fail to protect against other strains. Here we report that three recombinant rabies virus (RABV) vector vaccines encoding two copies of GPs covering both MARV lineages induced pseudovirus neutralizing antibodies in BALB/c mice. Furthermore, high-affinity human neutralizing antibodies were isolated from a humanized mouse model. The three vaccines produced a Th1-biased serological response similar to that of human patients. Adequate sequential immunization enhanced the production of neutralizing antibodies. Virtual docking suggested that neutralizing antibodies induced by the Angola strain seemed to be able to hydrogen bond to the receptor-binding site (RBS) in the GP of the Ravn strain through hypervariable regions 2 (CDR2) and CDR3 of the VH region. These findings demonstrate that three inactivated vaccines are promising candidates against different strains of MARV, and a novel fully humanized neutralizing antibody against MARV was isolated.

## Introduction

Marburg virus disease (MVD) is a severe and fatal viral haemorrhagic fever caused by the Marburg virus (MARV), and pathogen testing needs to be performed in a biosafety level 4 laboratory (BSL-4). The average fatality rate for MVD is about 50%, but fatality rate varies from 24 to 88% for different MARV strains [1,2]. Although the World Health Organization (WHO) declared the end of Uganda's MVD outbreak in 2017 [3], MVD outbreak reoccurred in Guinea in 2021, which was the first known case of MVD in West Africa [4]. Current research generally supports Egyptian fruit bats as the natural reservoir host of MARV [5–8]. Recent studies indicate that domestic pigs can be infected with another fatal filovirus, Ebola virus (EBOV), and spread it to humans [9–11]. The potential risk of domestic pigs to act as hosts for filoviruses raises concerns about the emergence of new filovirus diseases. Therefore, there is an urgent need to develop candidate vaccines and antibodies against MARV.

The MARV glycoprotein (GP) mediates attachment and entry into the target cells [12]. In the natural MARV structure, GP is a trimer on the virion surface. Each trimer comprises GP1 and GP2 subunits anchored together by a disulphide bond [13]. GP1 contains a receptor-binding core topped by a glycan cap and a heavily glycosylated mucin-like domain [14]. These two highly glycosylated domains block the GP1 subunit. The hyperglycosylated domain covers the epitope of GP1, which restricts access to putative receptor-binding sites and promotes viral immune evasion [15,16]. GP2 includes two heptad repeats and a transmembrane domain, which anchors GP to the viral membrane and triggers membrane fusion to enable virus entry. Filoviruses enter host cells through macropinocytosis, and after entering the endosome, the GP precursor protein is cleaved by furin and transported from the endoplasmic reticulum to the Golgi apparatus so that mucin-like polysaccharides and glycan are removed [17–21]. Then, the GP precursor protein is decomposed into two different subunits, GP1 and GP2, which are able to bind to the filovirus receptor Niemann Pick C1 (NPC1) [22]. Therefore, GP is the main target of MARV-neutralizing antibodies.

Flyak et al. isolated neutralizing antibodies against MARV from human survivors, and showed that neutralizing antibodies inhibit the virus by binding to receptor-binding sites (RBS) [23]. Bozhanova et al. analysed the human antibody variable gene repertoire using a computational approach called the position-specific structure scoring matrix (P3SM). They obtained a chimeric antibody that was completely analysed and designed in silico, based on the structure of the MR78 antibody described by Flyak et al., which neutralized the MARV Uganda strain in vitro [24]. Fusco et al. used mucin-deficient recombinant MARV GP as the immunogen and obtained 6 murine antibodies that neutralized Vesicular Stomatitis Virus (VSV)-based pseudovirus in vitro. Moreover, purified antibody 30G5 completely protected BALB/c mice after 1 h of challenge with MARV Ravn strain. This murine mAb was found to neutralize pseudoviruses by recognizing the MARV GP2-wing region instead of the RBS region [12]. Froude et al. immunized cynomolgus monkeys with viral replicon particles (VRP) expressing GP of the MARV Ci67 strain, and obtained four recombinant antibodies using a phage display library. Plaque reduction neutralization (PRNT) and VSV vector pseudovirus neutralization assays both demonstrated the in vitro neutralizing activity of the four antibodies. Antibody R3F6 fully protected IFN receptor knock-out mice 24 h after challenge with MARV Ci67 strain [25]. Marzi et al. reported a monoclonal antibody cocktail for MARV post-exposure therapy, which included one neutralizing and two non-neutralizing mouse anti-MARV antibodies, demonstrating that this cocktail provided 67%-100% protection after MARV infection in hamsters [26].

There are currently no licensed treatment or vaccine for MARV. Although remdesivir has been used in clinical studies to treat filovirus infections, the safety of remdesivir needs to be further verified due to severe adverse reactions when combined with other drugs [27–29]. Current MARV vaccine candidates, particularly immunogens based on Filoviridae such as MARV, failed to generate potent immune responses or neutralize antibodies [30–32]. In addition, cross-protection by vaccination between different MARV strains is challenging [33]. To address this issue, in this study, we selected three strains covering the two major MARV lineages and constructed three RABV vector-based inactivated vaccines encoding two copies of MARV GP. These three vaccines induced robust humoral and cellular immune responses in mice. In addition, we isolated 8 high-affinity antibodies from immunized humanized mice, including one neutralizing antibody.

## Materials and methods

### Biosafety and animal ethics statement

The packaging of the recombinant viruses, all cultures of viruses, the verification of virus inactivation, and the intracerebral injection of live viruses in suckling mice were completed in a Biosafety Level 2 Laboratory (BSL-2). All mice used in this study were handled strictly according to the recommendations described in the Chinese Ethical Guidelines for Laboratory Animal Welfare (GB 14925-2001). All mice were provided with adequate food and water. Animal experiments were approved by the Changchun Veterinary Institute of the Chinese Academy of Agricultural Sciences and the Animal Welfare and Ethics Committee of the Chongqing Academy of Animal Husbandry.

### Viruses, cells, and antibodies

The parental virus SRV9 was amplified in NA cells. Recombinant viruses were packaged and cultured using BSR cells. BSR, NA, and 293 T cells were cultured in DMEM medium (Gibco, C11995500CP) containing 10% fetal bovine serum (FBS; Gibco, 10099141) at 37°C. After inoculation with viruses, the serum concentration in the culture medium was reduced to 5%. The 293F cells were cultured and transiently transfected in fully synthetic serum-free OPM-293 CD05 medium (OPM Bioscience, 81075-001) at 37°C and 125 rpm, in a humidified atmosphere comprising 8% CO_2_. Primary mouse splenocytes were cultured at 37°C in RPMI-1640 medium (Sigma, R8758) containing 10% FBS and 1% penicillin–streptomycin (P/S; Gibco, 15140122). Colony-stage grown hybridoma cells were cultured in a semi-solid medium (StemCell, 03804). Mouse myeloma Sp2/0 cells and hybridoma cells were expanded in advanced RPMI-1640 medium (Gibco, 12633020) containing 10% FBS and 1% P/S.

Fluorescein isothiocyanate-conjugated anti-RABV N protein monoclonal antibody (800–092) and Anti-Rabbit IgG (H + L) antibody (BS10950) were purchased from Fujirebio and Bioworld, USA, respectively. Human monoclonal antibody MR191 (against MARV GP) was deposited in our laboratory (Changchun Veterinary Research Institute, Changchun, CAAS). HRP-conjugated goat anti-mouse IgG (H + L) antibody (BS12478) and goat anti-human IgG (H + L) antibody (BS10903) were purchased from Bioworld, USA. HRP-conjugated antibodies used to differentiate murine antibody subtypes IgG1 (1071-05), IgG2a (1081-05), IgG2b (1091-05), IgG2c (1078-05), and IgG3 (1101-05) were purchased from Southern Biotech Corporation, USA. Anti-human lambda light chain antibody (L1645), peroxidase antibody (A5175), anti-human kappa light chain antibody (K3502), and peroxidase antibody (A7164) were purchased from Sigma Aldrich, USA. HRP-conjugated mouse anti-human IgG Fab antibody (A01855) was purchased from GenScript, China.

### Generation and recovery of recombinant RABV vectors

To mimic the native structure of GP, we used the complete native sequence of MARV (including the signal peptide and transmembrane region) instead of the codon-optimized sequence (Supplementary Table 1) when constructing the three recombinant viruses. The SRV9 reverse genetic operating system was modified according to our previous description [34]. Briefly, RABV GP was replaced by MARV GP using the *Pst*I and *Kpn*I restriction endonuclease cleavage sites based on the cDNA of the parental virus SRV9. To increase the number of MARV GPs on the surface of the recombinant virus, we used the *Bsi*WI and *Pme*I cleavage sites to insert an addition copy of the MARV GP gene between the P and M genes of the parental virus. We constructed three full-length viral cDNAs containing the GP genes of different strains of MARV, named 2A, 2M, and 2R.

The full-length viral cDNA and support vectors carrying the RABV N, P, L, and G genes were combined to co-transfect BSR cells in 6-well plates using Lipofectamine 3000 transfection reagent (Invitrogen, L3000008). The transfected cells were observed every day to judge whether to exchange the culture supernatant with fresh medium (high-glucose DMEM containing 5% FBS). Seven days post-transfection, the cells were collected, frozen at −80°C, and the thawed cell debris and medium mixture was re-seeded into BSR cells. After repeated inoculation 3 times, the medium mixture was centrifuged at 5000 rpm and 4°C for 30 min to remove cell debris, and immunofluorescence (IF) was used to identify the production of recombinant virus. BSR cells were seeded with supernatant from each transfection well. After 72 h, the cells were fixed with pre-cooled 80% acetone solution at room temperature for 30 min. After washing the residual acetone solution with PBST, the FITC-conjugated anti-RABV N protein monoclonal antibody (Fujirebio, 800–092) was diluted with PBS solution containing 5% BSA and incubated with Evans blue (Sigma, E2129) at a final concentration of 0.02% (m/v) for 1 h at 37°C. After washing 3 times with PBST, the fluorescent lesions were observed under the microscope. The presence of fluorescent speckle aggregates indicated the recovery of infectious recombinant viruses. The virion suspension successfully reacting with anti-RABV N antibody was identified by immunofluorescence with homemade rabbit serum (1: 50) and an FITC-conjugated secondary antibody (1:200; Bioworld, BS10950) to ensure the expression of the GP of each strain. The recombinant virus expressing Angola strain GP was named rS-2A, the Musoke strain was named rS-2M, and the Ravn strain was named rS-2R.

### Virus inactivation and sucrose gradient purification

The rS-2A, rS-3M, and rS-2R vector viruses were used to infect BSR cells at MOI = 0.5 and cultured on a large scale. After culturing for 3 days, the virus liquid culture was placed at −80°C and frozen for 24 h. The virus solution was thawed on ice and centrifuged at 5,000 rpm and 4°C for 30 min to remove cell debris. The inactivator β-propiolactone (SERVA, 33672.51) was added at a ratio of 1:4000 to the virus suspension, mixed by inverting the vial, and incubated at 4°C for 24 h followed by incubation at 37°C for 2 h to hydrolyse β-propiolactone. The inactivated virus solution was centrifuged again, after which zinc acetate solution (1M, v/v = 1:50) was added to the supernatant to precipitate the viral particles. After standing at 4°C for 1 h, the solution was centrifuged again, and the pellet was suspended in a saturated EDTA solution of 1/80 of the original volume and dissolved at 4°C overnight. After centrifugation at 3,000 rpm and 4°C for 30 min to remove undissolved substances, the supernatant was collected as the concentrated solution. The virus solution was subjected to density gradient centrifugation (4°C, 21,000 rpm, 90 min) using sucrose solutions with concentrations of 20%, 30%, 40%, and 55%, respectively. White bands in the range of 40% to 55% were collected and centrifuged at 30,000 rpm and 4°C for 90 min to remove the sucrose solution. The resulting white pellet was resuspended with STE solution (pH = 7.5; Solarbio, T1110). The purified inactivated virus solution was stored at −80°C.

### Transmission electron microscopy (TEM) analysis

The purified inactivated virus solution was diluted 80 times with PBS and incubated with a 200 mesh copper-plated grid for 1 h at room temperature. Then, excess liquid was blotted from the edges of the mesh, after which the sample was negatively stained with 2% phosphotungstic acid solution (PTA) for 2 min and then air-dried. The observation was performed using a transmission electron microscope (JEM-1200EXII, JEOL) at an accelerating voltage of 80 keV and a magnification of 40,000 times.

### SDS-PAGE and western blot analysis

The purified virus particles were mixed with reducing SDS-PAGE loading buffer (CWBIO, CW0027S) and heated in a boiling water bath for 5 min. Virion proteins were separated using a 10% SDS-polyacrylamide gel. Coomassie brilliant blue (Beyotime, P0017F) staining was performed for 30 min and then de-stained overnight. The protein bands were transferred to nitrocellulose (NC) membranes for western blot analysis. The membranes were blocked with 5% skim milk in PBS for 2 h at room temperature, and incubated overnight at 4°C with a 200-fold dilution of MR191 monoclonal antibody in blocking buffer. After washing with TBST, the NC membranes were incubated with an HRP-conjugated goat anti-human secondary antibody (Bioworld, BS10903) for 1 h at room temperature. After washing, electrochemiluminescence (ECL) substrate (Beyotime, P0018S) was added dropwise, and the bands were captured using a chemiluminescence imager (Bio-Rad, ChemiDoc Touch).

### Virus titration

Use 96 deep-well plates to dilute the viral solution by adding 900 μL of DEME without FBS to each well. The first column of the deep-well plate was added with 100 μL of virus solution of unknown titre and mixed by repeated blowing using a 1 ml pipette tip. Take 100 μL of the mixed virus dilution, add that to the second column, and mix it in the same way. Each well was diluted 10 times and serially to 10^10^ times. Samples of different dilutions were added to 96-well cell culture plates, in the order of 10^1^–10^10^, with 100 μL per well repeated four times, and a blank cell control and a virus stock control were set up. 96-well cell culture plates were pre-inoculated with a density of 5 × 10^5^ cells/ml BSR cells at 100μl per well. The supernatant in each well was discarded after 72 h culture at 37°C 5% CO_2_ and washed twice with PBS solution. After washing, 150 μL of −20°C pre-cooled 80% acetone solution was added to each well and fixed at room temperature for 30 min. Wash 4 times with PBST solution at the end of the fixed. The FITC-conjugated anti-RABV N culture mAb was diluted 200-fold with PBST solution containing 5% BSA as the solvent and incubated for 2 h at 37°C for 100 μL per well. The PBST solution was washed five times, and then the fluorescent lesions were observed using an inverted fluorescence microscope. Viral titres were calculated using the Reed–Muench method based on the appearance of fluorescent foci in the viral solution at each dilution.

### One-step growth curves and genetic stability assay

The three recombinant viruses and the parental virus SRV9 were used to infect BSR cells at MOI = 0.5, and the viruses were collected every 24 h. The virus solution was 10-fold serially diluted in DMEM medium without serum and then seeded into BSR cells in 96-well plates. The cells were fixed with pre-chilled 80% acetone solution and stained with FITC-conjugated anti-RABV N antibody. Fluorescent lesions in each well were observed under a microscope to determine whether the virus solution at the corresponding dilution of the well was infectious. Virus titres were calculated using the Reed–Muench method based on the number of fluorescent foci at each dilution. The test was repeated three times for virus samples of unknown titre at each time point.

The recombinant virus was continuously amplified and cultured in BSR cells. The fifth and tenth-generation recombinant viruses were used to infect BSR cells for 48 h. Viral genomes in the culture were extracted (TIANGEN, DP315) and reverse transcribed into cDNA (TaKaRa, 6215A) according to the manufacturer's instructions. Polymerase chain reaction (PCR) was performed using the primers listed in Supplementary Table 1 and a high-fidelity polymerase (TaKaRa, R045B). The PCR amplification products were sequenced to assess the frequency of mutations in the MARV GP gene.

### Pathogenicity analysis

For the pathogenicity assay, 10^5^ TCID_50_ recombinant virus and parental virus were used for intracerebral infection of 3-day-old ICR suckling mice, and a PBS group was included as a negative control. Vaccinated suckling mice were continuously monitored (30 days) for survival and signs of disease such as ruffled fur, hunched back, and ataxia. Brain tissue was subjected to histopathological analysis after monitoring. Briefly, brain tissue was fixed in 10% formalin solution and embedded in paraffin. For histopathological evaluation, sections were stained with hematoxylin–eosin (HE; Solarbio, G1120).

### Immunization

The 6- to 8-week-old female BALB/c mice were randomly divided into 8 groups (*n* = 6/group), one group for each of the three recombinant viruses with/without adjuvant, an adjuvant control, and a PBS control group. Mice were injected intramuscularly with 20 μg of inactivated rS-2A, rS-2M, or rS-2R. Each virus was mixed with AddaVax adjuvant (InvivoGen, vac-adx-10)/PBS in an equal volume, yielding 100 μl (50 μl of each hind limb). All mice were vaccinated on days 0, 14, and 21. Serum was collected at the first, second, third, and fifth weeks after the immunization, and heated at 56°C for 30 min to inactivate the complement.

### Production of MARV GP protein

#### Based on the prokaryotic expression system

The GP genes of the three strains were amplified using the primers listed in Supplementary Table 1. The target fragment was ligated into the *E. coli* expression vector pET-30a(+) between the *Eco*RI and *Not*I restriction sites. The constructed expression vector was used to transform BL21 (DE3) chemically competent cells (TRANS, CD601), and kanamycin sulphate (Solarbio, K1030) was added for screening. After scale-up culture in liquid LB medium, IPTG solution (Solarbio, I1020) was added to a final concentration of 0.6 mmol/L, and protein expression was induced at 37°C and 200 rpm for 7 h. The bacterial cells were harvested by centrifugation and resuspended in binding buffer (20 mM sodium-phosphate, 10 mM imidazole, 8M urea, pH = 7.4). The cells were disrupted using an ultrasonic cell disintegrator (SCIENTZ, Scientz-IID). After centrifugation at 12,000 rpm for 15 min at 4°C, the supernatant was collected and incubated with His affinity chromatography medium (TRAN, DP101) at 4°C overnight. On the next day, the chromatography medium was washed 3 times with washing buffer (0.02 M PBS, 25 mM imidazole, 8M urea, pH = 7.4), and the protein was collected using elution buffer (20 mM sodium-phosphate, 500 mM imidazole, 8M urea, pH = 7.4). Proteins expressed as inclusion bodies were refolded using dialysis buffer (10 mM imidazole, 200 mM NaCl, 20 mM sodium-phosphate, 1 M urea).

#### Based on the mammalian cell expression system

The pCAGGS expression vector carrying the MARV GP gene and Strep purification tag was introduced into 293F cells. The cell density before transfection was 2 × 10^6^ cells/ml. The transfection dose of the expression vector was 1 mg/L. At7 days post-transfection, the 293F cells were resuspended in binding buffer (100 mM Tris-HCl, 150 mM NaCl, 1 mM EDTA, pH = 8) and protease inhibitor cocktail (M5293, AbMole) was added. After sonication, 11 U/mg of avidin was added and incubated in an ice bath for 30 min. Purification was performed using StrepTrap XT affinity chromatography medium (Cytiva, 29.401.328 AC) on a protein chromatography system (GE, ÄKTA Pure). The expression and purification of MARV Angola GP1 was conducted analogously, but 10×His tag is used, and Ni^2+^ metal-chelating medium (SUNRESIN, C415320101) was used for affinity chromatography.

### Enzyme-linked immunosorbent assay (ELISA)

Mice were anaesthetized with isoflurane at 7, 14, 21, and 35 days after immunization, and serum was collected through the submandibular vein. The three MARV GP proteins purified based on the prokaryotic expression system were resuspended in ELISA coating buffer (50 mM Na_2_CO_3_, pH = 9.6) at a concentration of 1.5 mg/L. The mixture was dispensed into 96-well ELISA high-binding microtitre plates (NEST, 514201) at a volume of 100 μl per well and bound overnight at 4°C. PBST solution containing 5% BSA was used as the blocking buffer and incubated at 37°C for 2 h, after which the microtitre plates were washed 3 times with PBST. The plates were incubated with serially diluted (3-fold) mouse serum in blocking buffer for 2 h at 37°C. After washing the plate, 15,000-fold diluted HRP-conjugated goat anti-mouse IgG (HL) antibody (Bioworld, BS12478) was added and incubated at 37°C for 1 h. The MARV GP-specific immunoglobulin subtypes in mouse serum were identified by incubation with HRP-conjugated IgG1, IgG2a, IgG2b, IgG2c, and IgG3 secondary antibodies (1:2000; Southern Biotech, 1071; 1081; 1091; 1078; 1101-05). After washing three times, 100 μl of tetramethylbenzidine (TMB) solution (Beyotime, P0209) was added to each well and incubated for 15 min at room temperature. Then, 50 μl of 2M H_2_SO_4_ was added to each well to stop the colour reaction. The absorbance was read at OD450 nm using a microplate reader (ThermoFisher Scientific, 51119180ET).

### Pseudovirus neutralization assay

The pcDNA3.1 expression vectors carrying the GP genes of the three MARV strains were used to express the GP for the pseudovirus. The pNL4-3.Luc.RE vector served as a pseudovirus backbone. A final concentration of 3 μg/well of donor plasmid and 3 μg/well of backbone plasmid were used to co-transfect 293 T cells in 6-well plates, and the three pseudoviruses were harvested 48 h later. The serum was heated at 56°C for 30 min and serially diluted in DMEM medium. Then, 100 μl of the sample and 50 μl of pseudovirus (except for cell controls) were added to 96-well white opaque plates (Beyotime, FCP968), including a virus control and cell control. After 1 h of incubation at 37°C, 50 μl of 293 T cells (4 × 10^5^ cells/ml) were added to each well and incubated for 48 h. After discarding 100 μl of supernatant per well, 100 μl of luciferase substrate (Beyotime, RG055M) was added to measure the relative light units (RLU). The percent neutralization was calculated based on luciferase activity, using the formula: neutralizing activity percent (%) = (RLU virus control-RLU sample) / (RLU virus control-RLU cell control) × 100%. The highest dilution at which the luciferase activity was reduced by more than 50% was taken as the neutralizing activity titre.

### Splenocyte proliferation assay

In each group, 3 mice were randomly selected and euthanized 7 days after the last immunization. Mouse spleens were collected, and the minced tissue was placed in a 70 μm cell mesh (Falcon, 352350) and thoroughly triturated using a 5 ml syringe plunger. The splenocytes were resuspended in RPMI-1640 medium (contains 1% P/S) and washed once by centrifugation at 1000 rpm and 4°C for 5 min. The spleen cell pellet was resuspended in red blood cell lysis buffer (Beyotime, C3702) and incubated at room temperature for 5 min. Then, 100 μl/well (2.5 × 10^6^ cells/ml) of the splenocyte suspension in RPMI-1640 medium containing 10% FBS and 1% P/S were seeded into a 96-well plate. Then, 10 μl of MARV GP protein (293F system expression and purification) at a concentration of 10 mg/L was added to each well except the control well. After 48 h at 37°C in a humidified atmosphere with 5% CO_2_, 10 μl of commercial Enhanced Cell Counting Kit-8 (CCK-8) reagent (Beyotime, C0042) was added to each well, followed by further culture for 4 h. The microplate reader (ThermoFisher Scientific, 51119180ET) was used to record the absorbance at OD450nm. The splenocyte proliferation index was calculated using the formula (OD stimulant-OD 1640) / (OD 1640-OD cell control).

### *Ex vivo* IFN-γ and IL-4 ELISpot assays

The differences in the secretion of IFN-γ and IL-4 in vitro by splenocytes of immunized mice were compared using the enzyme-linked immunospot assay. Three mice 7 days after final immunization were randomly selected from the adjuvanted experimental and the adjuvant-only control group and euthanized. The spleen cell suspension was prepared as described above, and 200 μl at a concentration of 2.5 × 10^6^ cells/ml was added to the ELISpot plate pre-coated with IFN-γ (Mabtech, 3321-4HPW-2) or IL-4 (Mabtech, 3311-4HPW-2). Then, MARV GP (from the 293F expression system) was added as a stimulus to the corresponding wells (10 μl/well, the concentration of 10 μg/ml) and incubated at 37°C in a humidified atmosphere with 5% CO_2_ for 36 h, during which the plate was not moved. After diluting the biotin-conjugated IFN-γ or IL-4 antibody (1:1000), 200 μl was added to each well, and it was allowed to stand at room temperature for 2 h. After washing 5 times with PBS, splenocytes were labelled with streptavidin-conjugated horseradish peroxidase (1:1000) and incubated at room temperature for 1 h. After washing the unbound peroxidase, TMB was used to develop the spots. Sterilized water was used to stop the colour reaction. The ELISpot reader (AID, Multispot reader Spectrum-iSpot) was used to read the number of spots.

### Cytokine detected by luminex

Mouse splenocytes were obtained by the method described above, and 2.5 × 10^5^ splenocytes were mixed with purified MARV GP (10 mg/mL, 293F expression system) and seeded in a 96-well plate. The supernatant was collected after culturing for 48 h, and cytokines were detected using a commercial Luminex kit (R&D Systems, LXSAMSM-11), according to the manufacturer's instructions. Luminex 200 (Luminex, LX200-XPON-RUO) was used to record the mean fluorescence intensity (MFI) information and perform standard curve fitting (five-parameter), with repeated collection 50 times for each test well and the doublet discriminator parameter set to 8000.

### Splenocyte surface marker staining

A total of 2.5 × 10^6^ splenocytes were mixed with purified MARV GP (10 mg/L, from the 293F expression system) were seeded into 12-well plates. After 72 h of culture, cells were collected by centrifugation and resuspended in PBS containing 2% FBS and 0.1% NaN_3_. Non-specific Fc receptors were blocked with 1 μg of anti-mouse CD16/CD32 antibody (BioLegend, 101301) per sample. Each sample was stained with 1 μg of APC-Cy7 conjugated anti-CD3e (ThermoFisher Scientific, 47-0031-82), 0.25 μg FITC-conjugated anti-CD4 (ThermoFisher Scientific, 11-0041-82), 0.25 μg PE-conjugated anti-CD8a (BioLegend, 100707), 0.5 μg APC-eFluor780 conjugated anti-B220/CD45R (ThermoFisher Scientific, 47-0452-82), 0.25 μg PE-conjugated anti-CD138 (BioLegend, 142504), 0.5 μg PE-Cy7 conjugated anti-CD80 (BioLegend, 104734), and 0.06 μg APC conjugated anti-CD62L antibody (ThermoFisher Scientific, 17-0621-82) for 40 min on ice. The fluorescence signal was recorded using a flow cytometer (BECKMAN COULTER, CytoFLEX).

### Immunization of antibody-humanized transgenic mice

The CAMouse [35] mice were subjected to a sequential immunization strategy. Immunizations were initially performed with inactivated BCG in 0.3% DMSO. The antigen for the two booster immunizations was the eukaryotic expression vector pcDNA3.1-Angola. Inactivated rS-2A (200μg) was mixed in equal volume with ISA 201 VG adjuvant (final volume = 200 μl), emulsified at room temperature, and injected subcutaneously at multiple points at 35, 49, 63, 77, and 91 days after primary immunization. The final immunization was based on subcutaneous injection of Angola GP expressed in the 293F system with ISA 201 VG adjuvant and splenocyte fusion 3 days later.

### Cell fusion and antibody screening

Single splenocyte suspensions were prepared as described above. The medium was changed to advanced RPMI 1640 (Gibco, 12633020). AO/PI dye (CountStar, RE010212) was used to determine the density and viability of splenocyte. The splenocytes and Sp2/0 cells were mixed at a ratio of 5:1. Electrofusion solution (BTX, 47-0001) was used to resuspend the mixed cells for cell fusion. The cell fusion instrument (BTX, ECM2001) was set to a voltage of 850 V and pulse of 40s. The fused cells were cultured in advanced RPMI 1640 medium (containing 10% FBS, 1% P/S) for 10 h (37°C, 5% CO_2_) for regeneration. Semi-solid medium (StemCell, 03804) was used for the culture of hybridoma cells for 10 days. Microscopically opaque cell colonies were picked using a 10 μl pipette tip and cultured in advanced RPMI 1640 medium containing HT supplement (Gibco, 11067-030). After 3–5 days, the supernatant of hybridoma cells with a confluence of 50–70% was collected for ELISA screening. GP1 at 0.5 mg/L was used for ELISA coating. The supernatant of hybridoma cells was used as the test sample. The secondary antibody (1:5000) was an HRP-conjugated mouse anti-human IgG Fab antibody (GenScript, A01855). The colour reaction was conducted at room temperature and terminated after 20 min.

### Analysis of antibody sequence and affinity

Double-antibody sandwich ELISA was used to identify isotypes of antibody light chains. Briefly, a 2000-fold diluted goat anti-human lambda light chain antibody (Sigma, L1645) and 1000-fold diluted goat anti-human kappa light chain antibody (Sigma, K3502) were used for ELISA coating. A 1000-fold diluted anti-human lambda light chains-peroxidase antibody (Sigma, A5175) and anti-human kappa light chain-peroxidase antibody (Sigma, A7164) were used for the detection of primary antibodies.

Total RNA from hybridoma cells was extracted using a DP430 Kit (Tiangen) according to the manufacturer's instructions. Each sample was eluted with 25 μl DEPC-treated water (Ambion, AM9915G). The 20 μl RNA samples were reverse transcribed into cDNA using a 6215A kit (TaKaRa). The final concentrations of the forward mixed primers and reverse primers listed in supplementary Table 3 were 50 μM. The PCR reaction was set up as follows (25 μl): 13 μl high-fidelity DNA polymerase (TaKaRa, R045B), 0.13 μl forward and reverse primers, 1 μl cDNA, 10.74 μl RNase-free water. The temperature program encompassed initial denaturation at 98°C for 10s, followed by 50 cycles of 98°C for 10s, 60°C for 15s, and 72°C for t40s, with a final elongation at 72°C for 5 min. The PCR products were analysed by 1% agarose gel electrophoresis and sequenced.

The Octet QK system was used to analyse the affinity of hybridoma cell culture supernatants to MARV Angola GP1 based on BLI technology (SARTORIUS, ForteBio). Kinetic buffer (1% BSA in PBST) was used to dilute all samples, and pure buffer served as a negative control. A His-tagged biosensor (SARTORIUS, HIS1K 18-5120) was used for detection after 20 min of hydration in the kinetic buffer. GP1 at 20 mg/L was immobilized on the sensor surface as the loaded antigen. The 2-fold diluted supernatant was used for detection and associated with the sensor. Data analysis was performed using ForteBio Data Analysis v11.0 and Data Analysis HT v11.0 software (ForteBio). Construction of high-affinity antibody light and heavy chain expression vector, and then transfected 293 T cells to verify the cross-neutralizing activity of the antibody.

### Data statistics and analysis

All experiments in the study were replicated at least 3 times and the results were expressed as Mean ± SEM. CytExpert software (version 2.4) was used to analyse flow cytometry data. JalView software (version 2.11.2.2) was used for antibody sequence alignment analysis and display. TBtools software (version 1.098746) was used to visualize the absorbance of hybridoma cell culture supernatants. Statistical analysis was performed using GraphPad Prism software (version 9.02). When analysing the statistical differences between two data groups, the sample's hypothesis type was judged according to the homogeneity of variance of the data (equal variance/heteroscedasticity). The interaction between the mean values of each dataset was considered in the case of three or more datasets. Single response variables were analysed using one-way ANOVA. Two-response variables were analysed using two-way ANOVA. Differences with *p*-values of less than 0.05 were considered statistically significant.

## Results

### Design of viral cDNA encoding two copies of MARV GP gene

**Figure 1.**
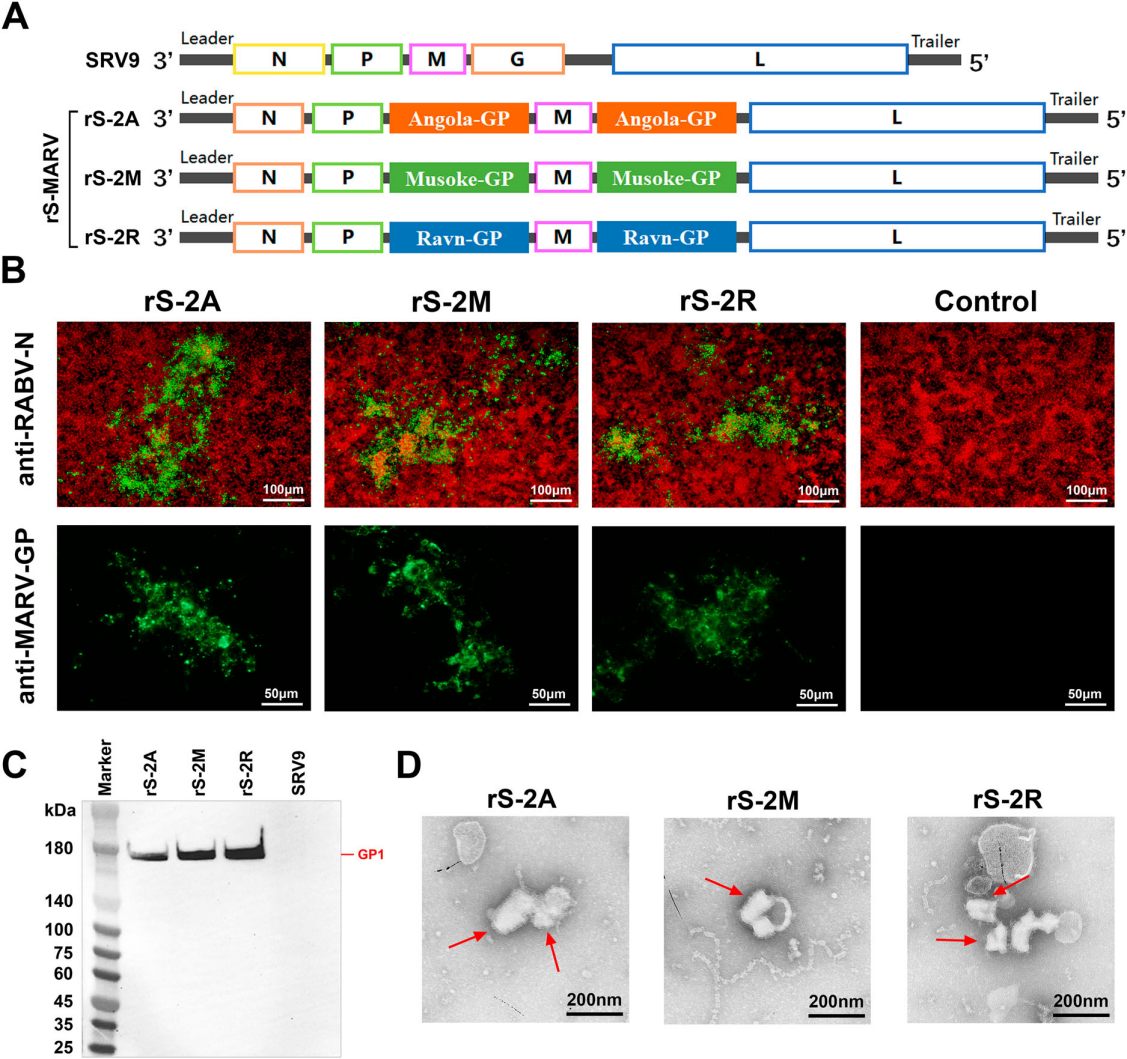
Construction and identification of recombinant rS-MARV virus. (A) The cDNA structures of the recombinant virus and parental SRV9 virus. The recombinant viruses encoding the two copies of MARV GP were named rS-2A, rS-2M, and rS-2R, respectively. The RABV GP gene was replaced with the MARV GP gene using the *Pst*I and *Kpn*I restriction sites. Another copy of the MARV GP gene was inserted between the P and M genes of the cDNA using the *Bsi*WI and *Pme*I restriction sites. The three recombinant viruses are collectively referred to as rS-MARV. (B) Identification of recombinant viruses by immunofluorescence. A sample comprising 1 ml of the third blind serial passage supernatant after transfection was seeded into a 24-well plate in which BSR cells were cultured. Pre-chilled 80% acetone solution was used for cell fixation and permeabilization. Anti-RABV N (monoclonal antibody) and anti-MARV GP antibody (self-made rabbit serum) were used for identification, respectively. Cell supernatants not inoculated with viruses served as negative controls. (C) Identification of MARV GP1 subunits by western blot. A sample comprising 20 μg of purified virus particles as subjected to polyacrylamide gel electrophoresis. MR191 was used as the primary antibody (recognizing MARV GP1), and a HRP-conjugated anti-human IgG antibody was used as the secondary antibody for western blot identification. (D) The morphological characteristics of recombinant viruses were analysed by TEM.

Transcription of rhabdoviruses starts from the 3’ end of the genome. Transcription first generates a short positive-strand RNA leader sequence and then sequentially generates mRNA molecules encoding N, P, M, G, and L proteins according to the direction of transcription. Before initiating the transcription of the following gene, RNA polymerase needs to complete the transcription of the previous gene and dissociate from the template before binding to the next initiation sequence [36]. Therefore, the transcription of the rhabdovirus genome exhibits an attenuation phenomenon. We exploited this attenuation feature of RABV genome transcription when designing the full-length cDNA of the virus. While the first copy of MARV GP was inserted instead of RABV GP, the second copy of MARV GP was inserted between P and M genes using the *Bsi*WI and *Pme*I cleavage sites, resulting in a full-length viral cDNA encoding two copies of MARV GP (Figure 1(A)). Despite the replacement of RABV G, the recovery of the recombinant virus can still be completed with the help of a support vector encoding the RABV G gene. Theoretically, the two-copy genome design allows more MARV GPs to be expressed and therefore more immunogenic [37–40].

### Generation of recombinant virus particles

T7 RNA polymerase-initiated support vectors facilitate homologous recombination of full-length viral cDNAs with genes encoding other structural proteins. The cDNA encoding two copies of MARV GP gene and the support vector under the control of T7 promoter and T7 RNA polymerase were co-transfected into BSR cells to complete the packaging of the recombinant virions. We identified the infection of the recombinant virus by immunofluorescence using an anti-RABV N antibody in infected BSR cells, finding that all three recombinant viruses recovered infectivity (Figure 1(B)). To verify the expression of the exogenous protein, a self-made rabbit serum against different strains of MARV GP was used for immunofluorescence identification. Cells infected with recombinant viruses showed visible fluorescent foci, indicating the expression of MARV GP (Figure 1(B)). Strain-specific MARV antibodies can bind to infected cells.

The protein level of the exogenous protein incorporated by the recombinant viruses is a better indicator that MARV GP was successfully expressed by rS-MARV. By SDS-PAGE of the sucrose gradient-purified rS-2A, rS-2M, and rS-2R, we detected MARV GP2 subunit at around 40 kDa, but not the GP1 subunit (Supplementary Figure 1). We used the anti-MARV GP antibody MR191 for western blot analysis and detected the presence of the GP1 subunit around 180 kDa (Figure 1(C)). These results suggested that the foreign proteins carried by the recombinant virus were expressed and correctly cleaved. Negative-stain transmission electron microscopy (TEM) shows that rS-2A, rS-2M, and rS-2R exhibited “bullet-like” structural morphologies of composite rhabdoviruses (Figure 1(D)). These results suggest that three recombinant rhabdoviruses each encoding two copies of MARV GP genes were successfully obtained.

### Evaluation of the replication characteristics and pathogenicity of recombinant viruses

**Figure 2.**
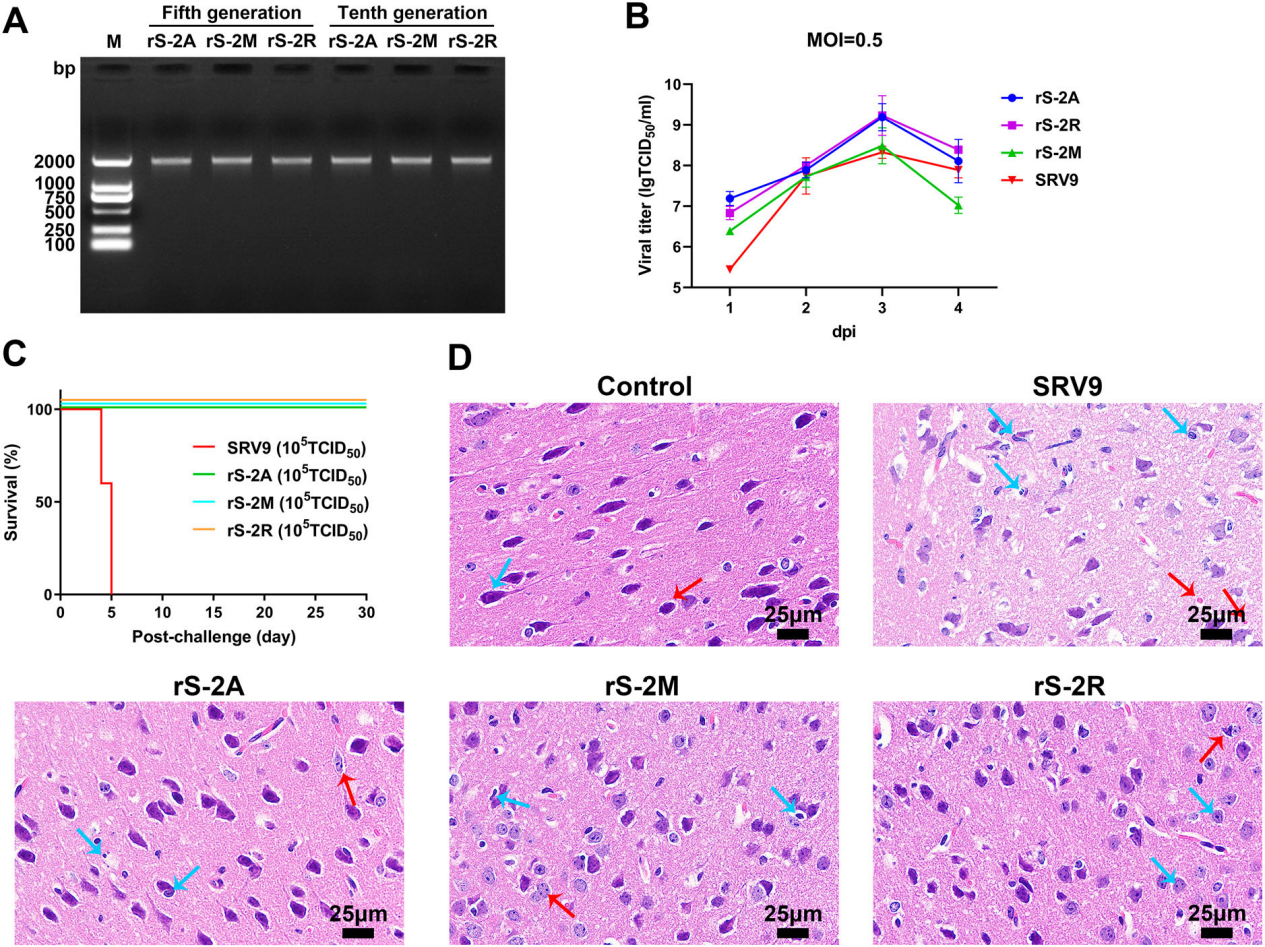
Preliminary evaluation of proliferation characteristics and safety of recombinant rS-MARV virus. (A) RT-PCR to identify the stability of MARV GP gene insertion in the recombinant virus. Reverse RNA transcription was performed using oligo dT and random 6nt primers. Sequencing analysis of RT-PCR products to determine whether the MARV GP gene can be stably inherited. (B) Growth kinetics of rS-MARV. BSR cells were infected with rS-MARV and SRV9 (MOI = 0.5), the supernatant was collected for four consecutive days, and the virus titre was determined using an FITC-conjugated anti-RABV N antibody. Each sample is repeated 3 times at each time point. (C) Pathogenicity evaluation of rS-MARV. SRV9 and rS-MARV were intracerebrally injected into 3-day-old ICR suckling mice, and the survival of suckling mice was recorded for 30 consecutive days. (D) Histopathological analysis. Representative histological images of vaccinated mouse brains infected with rS-MARV. All images were observed at 400x magnification. The images show significantly reduced neuronal degeneration in the brains of mice inoculated with rS-MARV compared to inoculation with the parental virus SRV9.

Recombinant viruses obtained based on reverse genetic technology may not have stable inheritance, resulting in reduced genetic stability and replication ability. In this part, we studied the properties of from these two aspects. RT-PCR and sequencing results showed that the 5th and 10th-generation of rS-MARV recombinant viruses stably carried the MARV GP gene (Figure 2(A)). All three recombinant and parental viruses were tested by infecting BSR cells at MOI = 0.5 to assess the replication capacity of rS-MARV. The one-step growth curve suggested that the growth trends of rS-MARV and SRV9 were similar, both reaching the threshold at 3 days post-infection (dpi) and starting to decline at 4 dpi. The titre changes of rS-2A and rS-2R were similar, while that of rS-2M decreased rapidly at 4 dpi. Notably, the overall titre increase of the parental virus SRV9 was lower than that of the recombinant viruses by 3 dpi (Figure 2(B)).

In rhabdoviruses, GP not only mediates viral entry into host cells, which makes it the primary target for neutralizing antibodies, but it also determines the neurotropic and pathogenic characteristics of the virus. Therefore, we initially evaluated the pathogenicity of the three recombinant viruses. Survival analysis showed that all 3-day-old ICR suckling mice with brain infection with SRV9 died within 5 days (including euthanized suckling mice). By contrast, all suckling mice inoculated intracranially with the same dose of recombinant virus (10^5^ TCID_50_) survived during the observation period (Figure 2(C)).

Histopathological analysis showed that the neurons in the cerebral cortex of the SRV9-infected mouse brain tissue were disordered, with large loose spaces and a large number of vacuoles in the brain tissue (Figure 2(D)). Necrotic nerve cells with deviated nuclei (Figure 2(D), red arrows), as well as degenerated neurons with inflammatory cell infiltration, hyperchromicity and shrunken nuclear shapes (Figure 2(D), blue arrows) were observed. By contrast, brain tissues from the rS-MARV injected mice were intact, with moderate pathological changes with neuronal degeneration and pyknosis (Figure 2(D)). Nuclear shrinkage (blue arrow) and diffuse/aggregated necrosis (red arrow) were occasionally seen in some neurons in the rS-2A and rS-2M groups. In the rS-2R group, neuronal nuclei were rarely shrunken, and some neurons had horn-shaped basophilic bodies (Figure 2(D)).

These results suggest that rS-MARV is relatively safe in mice, with survival rate as high as 100%. Although rS-MARV showed an excellent survival rate under the premise of 100% lethality of the parental virus, the replication and proliferation ability of rS-2A and rS-2M was higher than that of the parental virus. From the biosafety perspective, we inactivated the three recombinant RABV vector vaccines for the immunization studies hereafter.

### Inactivated rS-MARV induces a MARV-specific humoral immune response

**Figure 3.**
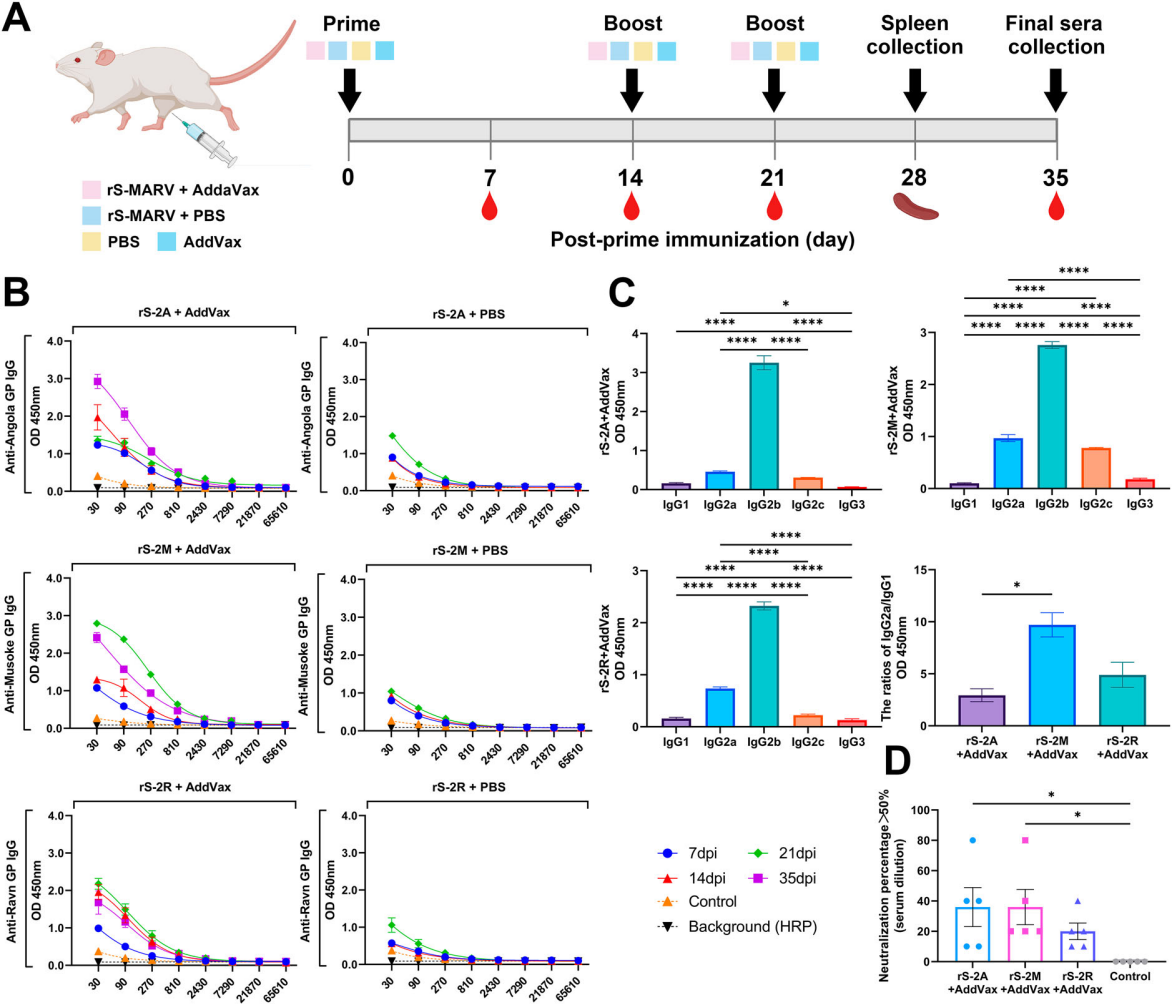
Humoral response to rS-MARV. (A) Immunization and experimental schedule. The abscissa represents the serum dilution ratio, and the ordinate represents the absorbance. The BALB/c mice (*n* = 6/group) were immunized with 20 μg of inactivated rS-MARV suspended in AddaVax adjuvant. The vaccine was injected into the hindlimb muscle of the mice. All mice were primed on day 0 and boosted on days 14 and 21. The minimum dilution of serum is 20. (B) Three sera recovered from immunized mice were randomly selected and diluted at 1:30 to prevent matrix effects. Indirect ELISA analysed serial 3-fold dilutions of serum samples to test the relative amounts of MARV GP-specific antibodies. Serum samples were tested 14 days after the final immunization (35 dpi) to characterize the persistence of the antibody response. The OD_450_ values at each dilution factor were fitted to a 4-parameter nonlinear equation to facilitate observation of trends. Negative control is PBS group. (C) The IgG isotype response of immunized mice was assessed by indirect ELISA 21 days after vaccination to assess Th1 versus Th2 biased humoral immunity. (D) Mouse sera were analysed using a lentiviral pseudovirus carrying a luciferase reporter gene. The 20-fold diluted mouse serum was used as the initial concentration for the pseudovirus neutralization assay. Neutralizing activity was defined as a reduction of luciferase activity by more than 50%. The image shows the neutralization titre of mouse sera sampled at 21 dpi. Negative control is PBS group. The minimum dilution of serum is 10.

Squalene is easily metabolized by the body, and numerous studies have shown that squalene-based oil-in-water nano adjuvants can induce both good humoral and cellular immune responses [41–43]. Moreover, the squalene adjuvant MF59 has been approved for influenza vaccines [44]. Therefore, we used AddaVax adjuvant, similar in composition to MF59, to evaluate the immunogenicity of inactivated rS-MARV. To assess immune responses elicited by the three inactivated recombinant virus vaccines, BALB/c mice (*n* = 6/group) were intramuscularly injected with 20 μg of inactivated recombinant virus mixed with AddaVax or PBS at the same ratio (v/v), and serum was collected from the mice according to the immunization procedure (Figure 3(A)). MARV-specific IgG in the serum was characterized by ELISA using soluble GP protein expressed in a prokaryotic system (Figure 3(B,C)). Mice immunized with rS-MARV plus adjuvant showed strong seroconversion with immunoreactivity to MARV GP, whereas no MARV GP-specific titres were detected in the PBS control group (Figure 3(B)). To confirm the dominance of Th1 and Th2 immune responses, we assessed isotype-specific antibody responses in mouse sera at 35 dpi. Based on the relative serum titres of IgG2 and IgG1, the immune response appears to have been biased towards Th1. At the same time, the rS-2M group had a stronger Th1 tendency than the rS-2A group (Figure 3(C)).

### Inactivated rS-MARV induced weak and non-persistent neutralizing antibodies

The ELISA results have proved that inactivated rS-MARV could induce the production of MARV GP-specific antibodies in mice, but whether it can induce neutralizing antibodies was still unclear. Accordingly, we tested the serum samples for the presence of specific antibodies with *in vitro* neutralizing activity against a lentiviral pseudovirus expressing a luciferase reporter gene. We tested four batches of mouse sera collected after immunization, but only weak neutralizing activity was detected at 21 dpi (Figure 3(D)), and three serum samples from each adjuvanted experimental group have one sample could not neutralize pseudovirus. These results suggest that the production of neutralizing antibodies requires additional immunization with a booster dose. Nevertheless, boosters did not produce neutralizing antibodies in all mice, and these low neutralizing antibody titres also did not appear at all times after immunization.

### Specific cellular immune responses induced by inactivated rS-MARV

**Figure 4.**
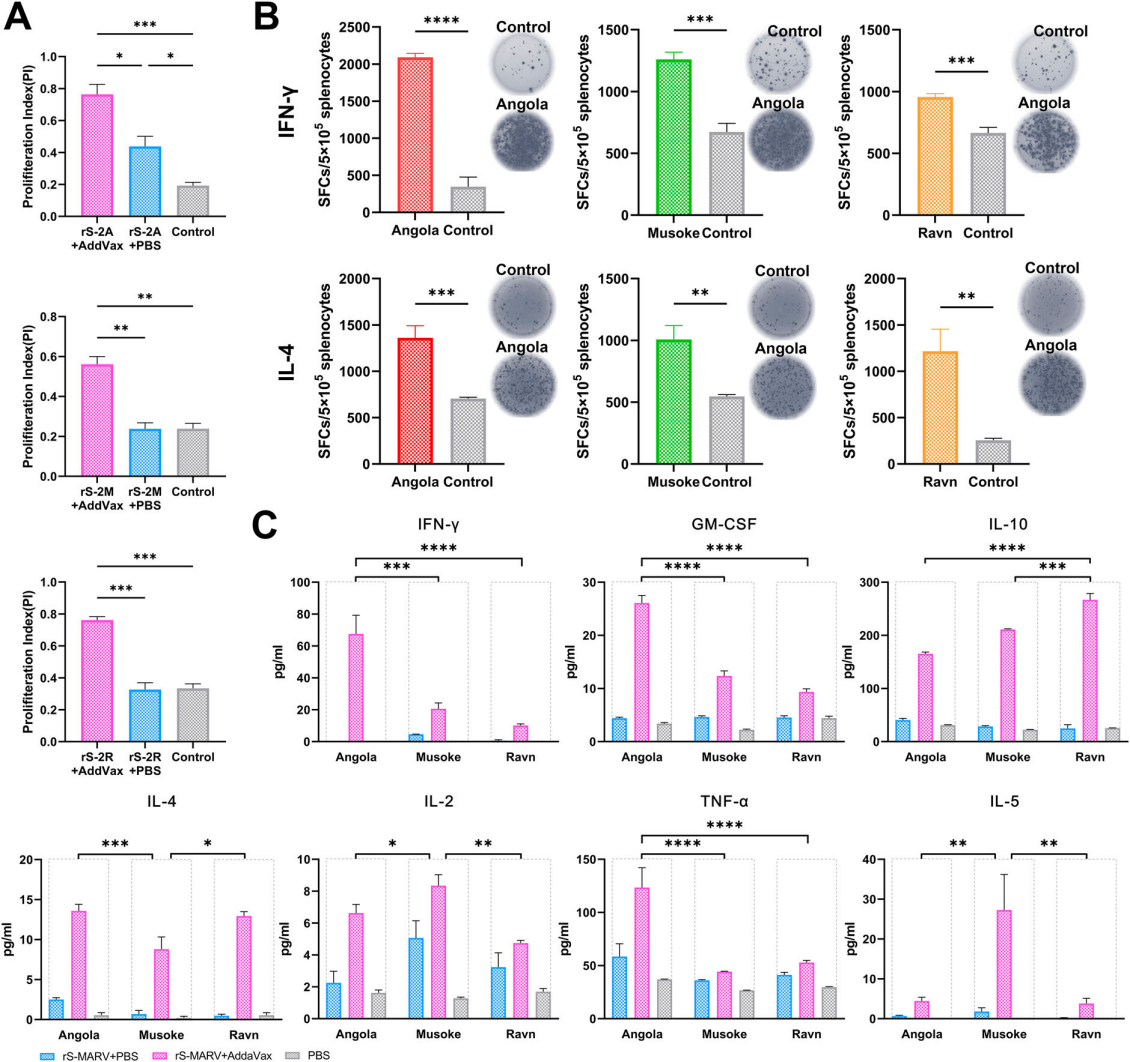
Cellular immunologic response to rS-MARV. One week after the last immunization, mouse spleens (*n* = 3/group) were collected, and spleen cells were re-stimulated with purified MARV GP. The stimulator concentration was 10 μg/ml, 10 μl per well of a 96-well plate. All samples were replicated 3 times, and data are presented as means ± SEM for each group. (A) Specific splenocyte proliferation. The CCK-8 assay was used to calculate the proliferation index. AddaVax-vaccinated mice served as negative controls. (B) The levels of IFN-γ and IL-4 secreted by splenocytes were measured using the ELISpot assay. The abscissa indicates the corresponding protein stimulus. AddaVax-vaccinated mice served as negative controls. (C) The Luminex method was used to determine cytokine levels in the culture supernatants of splenocytes. The differences in the specific secretion of cytokines by splenocytes after rS-2A, rS-2M, and rS-2R immunization were compared under the premise of considering each rS-MARV grouping. The abscissa indicates different MARV GP stimuli. Angola corresponds to rS-2A, Musoke corresponds to rS-2M, and Ravn corresponds to rS-2R. The fluorescence signals of the standards were fitted to a 5-parameter curve in Luminex200 software. $R_{IFN-\gamma }^{2}$ = 0.961, $R_{GM-CSF}^{2}$ = 0.992, $R_{IL-10}^{2}$ = 0.998, $R_{IL-4}^{2}$ = 0.926, $R_{IL-2}^{2}$ = 0.950, $R_{TNF-\alpha }^{2}$ = 0.995, $R_{IL-5}^{2}$ = 0.989, 3 decimal places.

The level of specific splenocyte proliferation was measured one week after the last immunization (CCK-8 method) to indirectly assess the phenomenon of vaccine-induced lymphocyte transformation (Figure 4(A)). We used the three MARV GPs expressed and purified using the 293F system as specific antigenic stimulators to avoid thymus-independent antigenic (TI-Ag) stimulation of splenocytes by residual endotoxin or lipopolysaccharide and other components of the prokaryotic expression system. Figure 4(A) shows that the proliferation level of splenocytes in mice immunized with AddaVax + rS-MARV was significantly higher than in mice immunized with pure inactivated virus and adjuvant controls. These results suggest that the vaccine induced the production of MARV GP-dependent splenocytes in mice.

In addition to the CCK-8 method which indirectly reflects the proliferation by detecting the metabolic activity of the cells. We performed ELISpot to monitor the proliferating and differentiated splenocytes captured by the corresponding cytokine antibodies. The number of spot-forming cells secreting IFN-γ and IL-4 was significantly higher among splenocytes from mice immunized with the adjuvant and antigen mixture than that in the adjuvant control group (Figure 4(B)). These results suggest that all three antigens, rS-2A, rS-2M, and rS-2R, were able to specifically induce splenocytes to produce IFN-γ and IL-4, and the proliferation of these lymphocytes is dependent on MARV GP of different strains.

### Inactivated rS-MARV enhanced specific T cell immune responses

The results of the Luminex assay showed that among the 7 cytokines detected, the rS-MARV with adjuvant all led to stronger cytokine secretion under the stimulation of the GP of the corresponding strain. Among the cytokines secreted by Th1 cells (IFN-γ, IL-2, TNFα), the ability of rS-2A to induce the secretion IFN-γ and TNFα was the highest among the three rS-MARVs, and the difference was statistically significant. The IL-2 level of rS-2M was significantly higher than that of the two other immunogens. Among Th2 cytokines (IL-4, IL-5, IL-10), rS-2M induced significantly lower IL-4 and higher IL-5 levels than rS-2A and rS-2R. In addition, the content of GM-CSF induced by rS-2A was significantly higher than that of rS-2M and rS-2R (Figure 4(C)). This may suggest that the AddaVax adjuvant helps activate APCs, including macrophages, and enhances the immune function of these cells. In addition, the overall trends of IFN-γ and IL-4 were consistent with the results of ELISpot analysis. These data suggest that adding AddaVax adjuvant can enhance Th1 and Th2 immune responses. The three rS-MARVs induced balanced secretion of Th1 and Th2 cytokines. Furthermore, the ability of MARV GP from different strains to stimulate the body to produce an antigen-specific cellular immune response was not the same.

**Figure 5.**
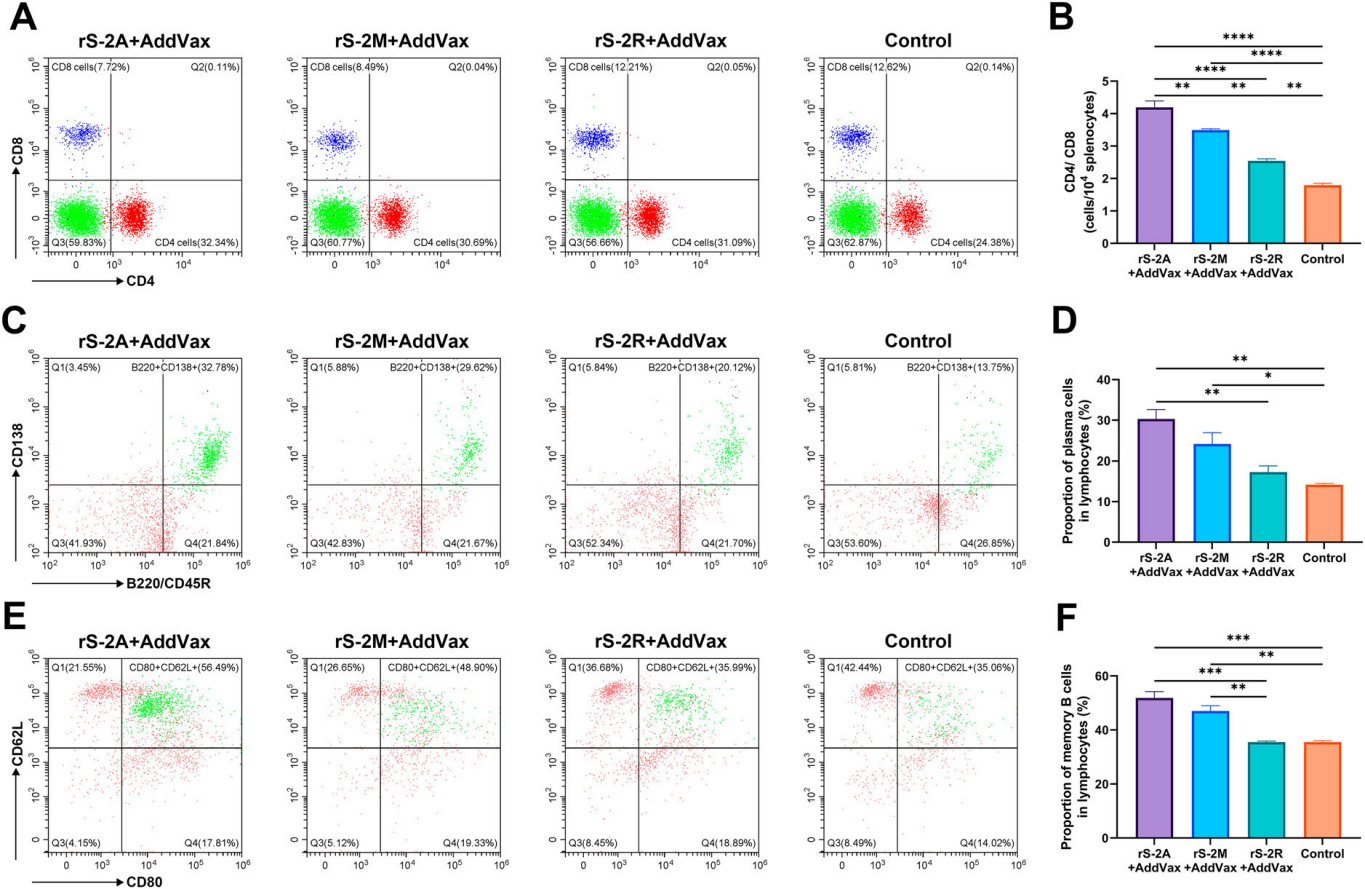
MARV-specific lymphocyte activation. Activation of lymphocytes induced by the inactivated virus vaccine was assessed by flow cytometry, including effector T cells (A,B), plasma cells (C,D), and memory B cells (E,F). All splenocytes were isolated on day 28 after primary immunization. The purified protein corresponding to the vaccine was used as a stimulus. Splenocytes of mice inoculated with AddaVax were included as a negative control. (A,C,E) show representative plots of lymphocyte populations from each group. (B) The CD4^+^ and CD8^+^ T cell ratio in the total lymphocyte population reflects the T cell immune response. (D,F) compare the proportion of plasma and memory B cells to total B cells.

To compare the ability of different strains to induce T cell immune responses, we analysed the number or proportion of T cell subsets among all lymphocytes by flow cytometry. The ratio of CD4 to CD8 was more than 1 in all experimental and adjuvant control groups, implying that MARV GP preferentially activated Th cells over suppressor T (Ts) cells. As can be seen in Figure 5(A,B), rS-2A with AddaVax adjuvant induced the highest cellular immunity level, followed by rS-2M and rS-2R. Nevertheless, the lowest level of rS-2R was still significantly higher than that of the adjuvant control group, indicating that all three rS-MARVs could promote T cell activation.

### Inactivated rS-MARV enhanced B cell differentiation

Figure 5(C,D) shows that after in vitro stimulation, all three rS-MARVs induced B cells to differentiate into plasma cells, which were significantly more abundant in the rS-2A and rS-2M groups than in the controls. Although there was only a statistically significant difference between rS-2A and rS-2R, the overall trend in Figure 5(D) indicates that rS-2A induced the strongest B cells differentiation.

Next, we simultaneously compared the proportion of memory B cells among lymphocytes in different groups to assess the potential of rS-MARV as a MARV vaccine candidate. While rS-2A and rS-2M induced memory B cells, there was no difference between rS-2R and controls (Figure 5(E,F)), suggesting that the number of memory B cells generated after rS-2R immunization was insufficient.

### A sequential immunization strategy elicited neutralizing antibodies in humanized mice

Low titres of neutralizing antibodies were detected in the serum of BALB/c mice 21 days after primary immunization with rS-MARV. Based on this finding, we attempted to isolate these MARV-specific antibodies with neutralizing activity.

**Figure 6.**
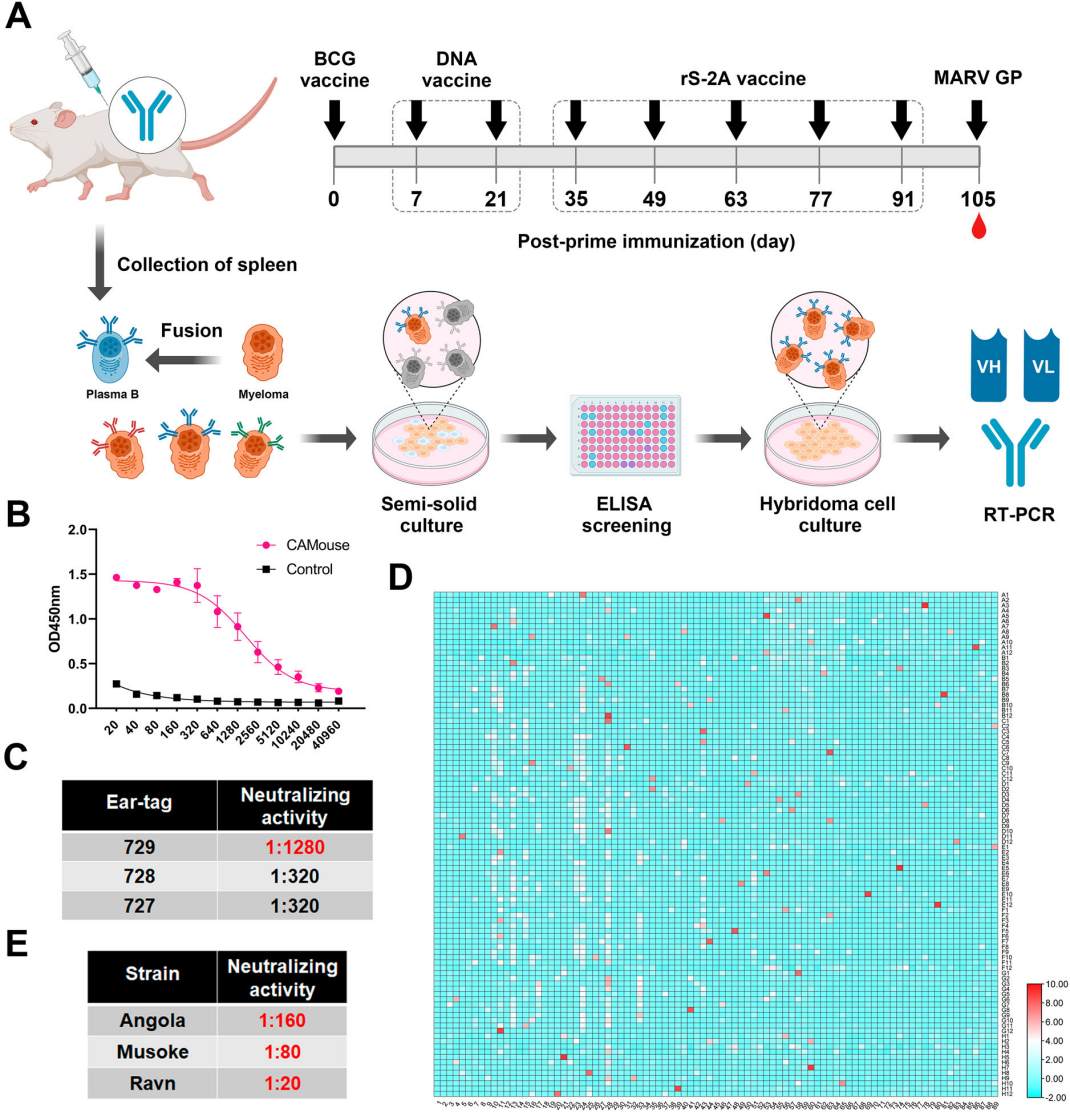
Discovery of fully humanized monoclonal antibodies against MARV. (A) Sequential immunization strategy of antibody-humanized transgenic CAMouse mice. Each mouse was inoculated with 100 μg of inactivated BCG to mobilize the immune system of the mice on day 0. DNA vaccine was used for primary sequential immunization, and inactivated rS-2A was used as a booster. Final stimulation with MARV GP was performed 3 days before cell fusion (105 dpi). (B,C) show mouse-specific IgG titres and neutralizing antibody titres at 105 dpi, respectively. The abscissa of (B) is the serum dilution. (D) Indirect ELISA screening of the culture supernatant of 8544 hybridoma cell lines. The abscissa indicates the plate number, and the ordinate indicates the well number. The response value is the absorbance at 450 nm (OD_450_). The heatmap is normalized, and red indicates a high absorbance value. (E) Pan-pseudovirus neutralizing activity of 60H07 against different MARV strains. 60H07 is expressed by 293 T cells.

Because the neutralizing antibodies induced by the inactivated vaccine rS-MARV in mice were weak and not persistent, we changed the dose and number of immunizations. As shown in Figure 6(A), we achieved high titre or long-lasting neutralizing antibodies that were not induced by the initial rS-MARV regimen using inter-vaccination of immunogens by different technical routes. Thus, rS-2A generated the most plasma and memory B cells after immunization, which provided more possibilities for the isolation of neutralizing antibodies.

### High-affinity fully humanized monoclonal antibody induced by inactivated rS-2A

To obtain humanized anti-MARV antibodies, we used transgenic mice (CAMouse) as an animal model to obtain anti-MARV monoclonal antibodies with neutralizing activity through hybridoma technology. The CAMouse is a transgenic mouse in which human immunoglobulin heavy chain and light chain genes have been inserted, while the endogenous mouse heavy chain and light chain genes have been deleted [45].

After the sequential vaccination, GP-specific antibodies and neutralizing antibodies have been detected in CAMouse on day 105 (Figure 6(B,C)). Previous studies suggests that MARV antibodies with neutralizing activity recognize only the RBS of GP. Accordingly, we used GP1 protein-containing RBS as the coating of ELISA to test the antibody titre and screen the hybridoma cell lines. False-positive results of ELISA caused by the immunogen as the coating protein were avoided as much as possible. From 8544 fused hybridoma cells, we screened 88 hybridoma cell lines (Figure 6(D)) whose absorbance (OD_450_) was more than 6 times higher than that of the negative control. These cells were transferred from 96- to 24-well plates for expansion, and 86 of the cell lines could grow normally. For further screening, we measured the affinity of the antibodies in the culture supernatant using ForteBio, a BLI-based macromolecular interaction instrument. The His-tagged GP1 protein was loaded onto the HIS1K biosensor, and the affinity of the antibody was detected by immobilizing the antigen. As shown in Supplementary Table 2, the *K_D_* values of the samples were between 10^−5^ and 10^−9^. We finally obtained 8 hybridoma cell lines (41G08, 42F04, 43E07, 43F07, 44F07, 49E08, 57A07, and 60H07) that can secrete high-affinity antibodies (*K_D _*= 10^−9^, nanomolar level) (Supplementary Figure 2), which were further expanded into 100 mm cell culture dishes. The neutralizing activity of the 20-fold concentrated supernatant was tested using a lentiviral pseudovirus based on the Angola strain. Surprisingly, antibody secreted by cell line 60H07 was able to neutralize the pseudovirus. For further scale-up, the light and heavy chain expression vectors were used to transiently transfect HEK293T cells. The neutralizing activity of the 60H07 harvested from the supernatant was 1:160 for the Angola strain, 1:80 for Musoke, and 1:20 for Ravn. This suggests that 60H07 has cross-neutralizing activity (Figure 6(E)).

### Essential characteristics of high-Affinity human anti-MARV-neutralizing antibodies

**Figure 7.**
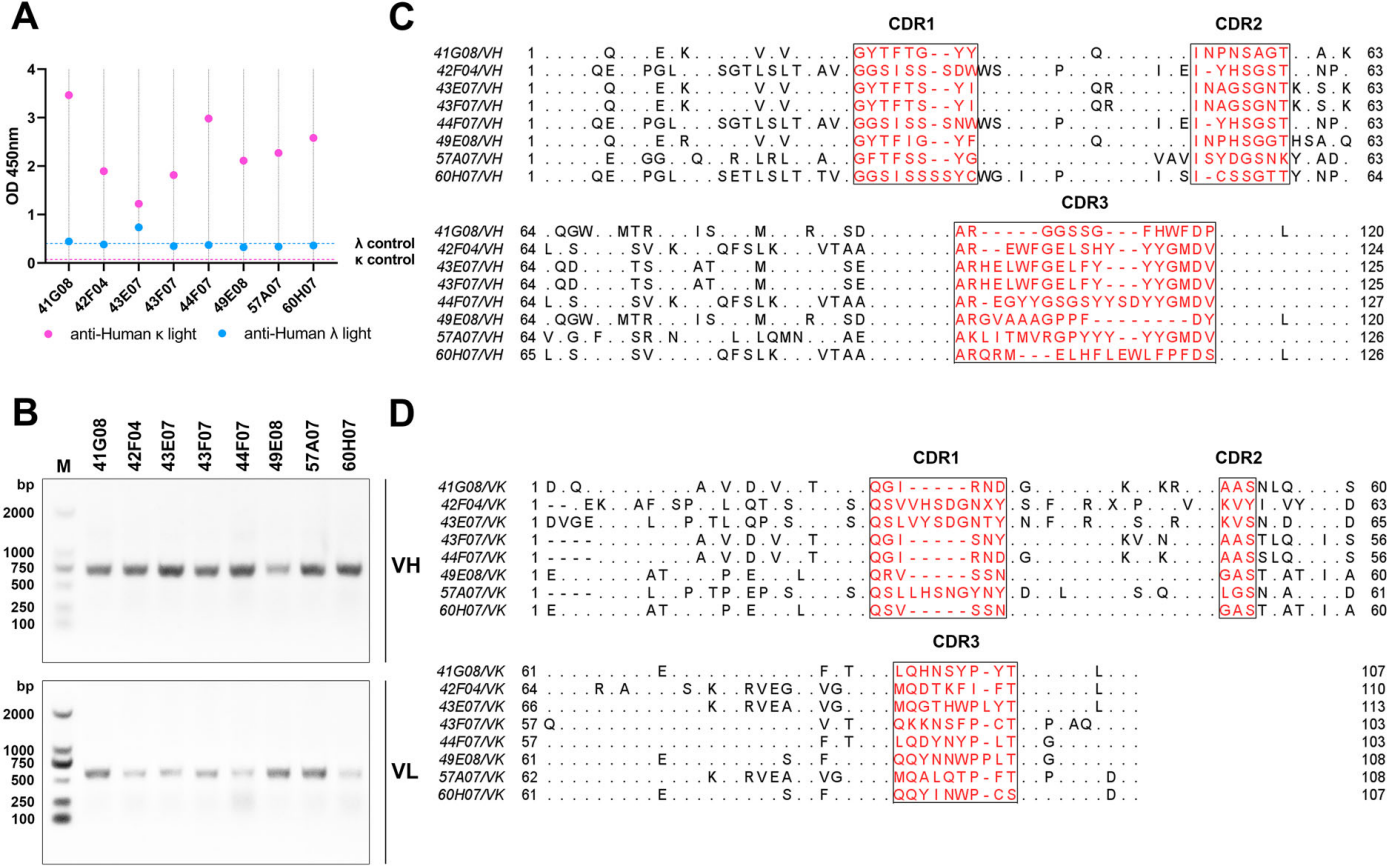
Properties of fully humanized MARV antibodies obtained from transgenic CAMouse mice. (A) Identification of antibody light chain isotypes. Double-antibody sandwich ELISA was used to differentiate antibody light chain isotypes. (B) RT-PCR amplified antibody sequences. Total RNA from hybridoma cells was extracted and reverse transcribed into cDNA. Multiplex PCR was used to amplify antibody VH and VK sequences. The VH band was approximately 740 bp, and the VK band was approximately 630 bp. (C,D) show the amino acid sequences of antibody VH and VK chains, respectively. The antibody's CDRs are marked in red, and the framework regions (FRs) show differential amino acids.

The subtypes of antibody light chains in the supernatants of 8 the hybridoma cell lines were identified by double-antibody sandwich ELISA. The results showed that all 8 antibodies had κ-type light chains (Figure 7(A)). We extracted the total RNA from the hybridoma cells and performed RT-PCR to obtain the underlying gene sequences of these antibodies. The primers in Supplementary Table 3 were used for multiplex PCR. We mixed the forward primers at a concentration of 50 nM and obtained the final amplification product (Figure 7(B)) through a single PCR reaction, which was used for Sanger sequencing. Figure 7(C,D) shows the amino acid differences of VH and VK of the eight antibodies, respectively.

**Figure 8.**
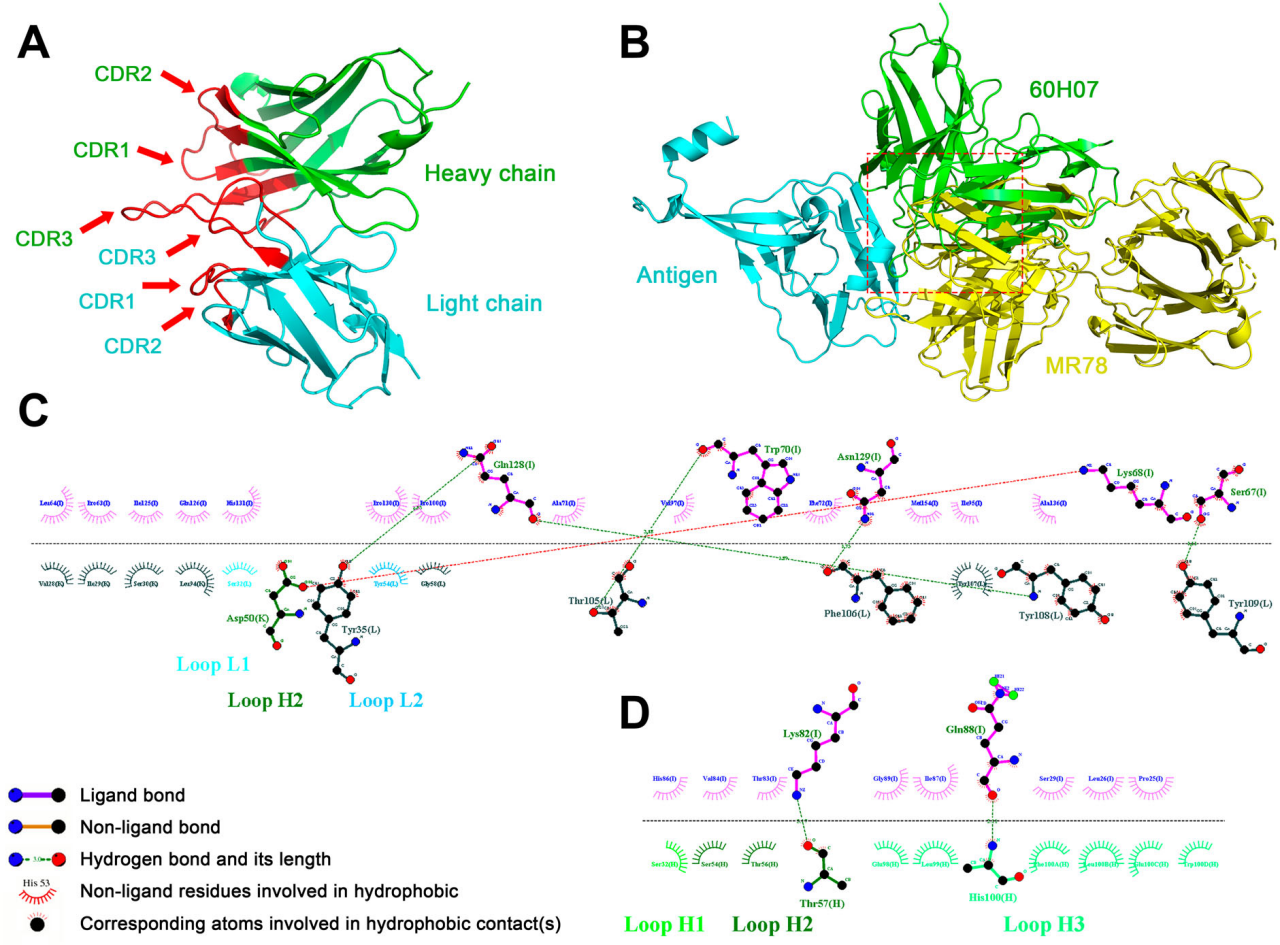
Simulated docking of antibody 60H07 with the Ravn GP antigen. The structure of 60H07 was predicted using the IgFold algorithm. The HADDOCK method was used to simulate antigen-antibody binding, and the RBS region was used as the approximate action site for docking with default parameters. The model with the highest score was used for subsequent analysis. Binding residues were predicted using the LigPlus algorithm. (A) Structural model of antibody 60H07. The spatial structure of the CDRs is shown in red. Green and blue are used to distinguish between heavy and light chains. (B) Spatial docking model of 60H07 with MARV GP. Comparison with known antibody-binding epitopes of MARV. The red box highlights the overlapping binding positions of 60H07 and MR78 to GP. (C) The detailed site of MR78 binding to GP was based on a published crystal structure (PDB ID: 5UQY). (D) Key predicted site of 60H07 binding to Ravn GP.

The 3D structure of the VH and VK of the only neutralizing antibody 60H07 was predicted using the IgFold algorithm, and the antibody model with the highest systematic score was used for docking with MARV GP protein. The CDR region of the antibody model was simulated to bind near the RBS site of the GP protein to find key amino acid sites. All other proteins were simulated using known structures to increase the confidence of the model predictions, except 60H07, which was predicted by the algorithm. The complex structure (PDB: 5UQY) of MARV GP bound by neutralizing antibody MR78, which was isolated from a human infection survivor, was used as a reference for the RBS-binding site. Figure 8(A) shows the spatial structure model of 60H07. The docked 60H07-GP complex exhibited an overlapping structure with the MR78-GP complex (Figure 8(B)), which suggests that 60H07 may play a neutralizing role by recognizing amino acid sites on GP similar to those recognized by MR78. To identify the specific positions of these amino acids, we analysed the hydrogen bonding and hydrophobic interactions between 60H07 and MARV GP. We found that despite the overlap of the docked 60H07-GP complex with MR78-GP, there were notable differences between the hydrogen-bonding residues of the CDR regions that bind the GP. Figure 8(C) shows the critical amino acid interactions between MR78 VH and GP in the MR78-GP complex crystral structure (ref. Hashiguchi et al.). The VH CDRs of MR78 bind the GP through non-ligand bonds (CDR2) and hydrogen bonds (CDR3), while the VH of 60H07 is wholly bound to GP through hydrogen bonding (Figure 8(D)). This single-energy binding is less stable than the MR78-GP complex. Nevertheless, it is worth noting that 60H07 was induced by an immunogen based on the Angola strain, while the MARV GP in the crystal structures are all based on the Ravn strain. Therefore, we speculate that the neutralizing ability of 60H07 has higher affinity for Angola GP.

## Discussion

There are no vaccines or antibodies approved for the clinical treatment of MVD. Although scientists hope to design ideal vaccines that can provide complete protection from different isolates or mutants, research on the Venezuelan equine encephalitis virus replicon showed that the replicon vaccine expressing the GP of the Musoke strain protected cynomolgus monkeys against lethal homologous challenge [33] but not against Ravn [46]. Most current studies on MARV vaccines do not test the immune effects of different strains. To this end, we designed three immunogens covering the two major lineages of MARV, hoping to inspire scientists to pay attention to the immune response to different strains of MARV.

The current MARV strains are divided into a branch that includes the Ravn strain, isolated in Kenya in 1987, together with the Durba strain, discovered in the Democratic Republic of Congo in 1999, and a second branch including the Angola strain with the highest fatality rate and the classic *Lake Victoria Marburg Virus* Musoke strain [47]. Notably, although there is only one serotype of MARV, amino acid differences between the two genetic lineages may affect the ability of vaccine candidates to provide cross-protection against different strains. Among all isolates, the amino acid differences between the Ravn and other strains were as high as 21–23% [48,49]. The fact that the Angola and Musoke strains are in the same genetic lineage may explain the cross-protection between the two induced by many MARV vaccines that use one of them as the antigen. However, the cross-protection effect against Ravn strains with lower amino acid homology differs. We used the RABV reverse genetic operating system to construct and evaluate three candidate vaccines encoding two copies of GP genes, and isolated antibodies with cross-neutralizing activity from fully humanized mice immunized with the Angola strain vaccine.

Almost all replicable recombinant viruses based on RABV vectors contain foreign genes inserted into the original genome [50–53]. This is done because the viral vector lacking the original GP is deficient in the packaging of recombinant viruses that can replicate and multiply [54]. In this study, we overcame these difficulties and describe the construction, reproductive properties, and immunogenicity of three recombinant viruses encoding two copies of MARV GP. We replaced RABV GP with MARV GP and inserted another copy of MARV GP between the P and M genes in the original viral cDNA. The results showed that rS-MARV induced specific humoral and cellular immune responses in mice. Moreover, neutralizing antibodies against three strains were detected at 21 dpi, albeit briefly. Notably, neutralizing antibody responses to MARV in human survivors were also limited [55,56]. At the same time, MARV induced the expression of IL-2, IFN-γ, and TNF-α but not IL-4. This Th2-imbalanced and strongly Th1-biased humoral immune response is similar to our results. The IgG2 subtype dominated the specific antibodies produced by mice immunized with rS-MARV. Furthermore, we focused on seven cytokines related to adaptive immunity and Th cell differentiation, including IFN-γ, GM-CSF, IL-10, IL-4, IL-2, TNF-α, and IL-5. The analysis of secreted cytokines and ELISpot results were mutually congruent and indicated a Th1 response. Perhaps the weak neutralizing antibodies produced by BALB/c mice may be attributed to these Th1-biased IgG2 antibodies against MARV GP. All the above results suggest that the antibody response generated by inactivated rS-MARV is consistent with the characteristics of human infection.

The surface of filoviruses contains only GP, which is cleaved by cathepsins after entering the body to remove the mucin-like domain and glycan cap, after which it can bind to the NPC1 receptor. Nevertheless, MARV does not depend on cathepsin [13]. Although the mucin-like domain of EBOV immediately follows GP1 and terminates before the cleavage site, it spans GP1 and GP2 in the MARV structure. The different positions of the mucin-like domains will result in different exposed surfaces of the GP, resulting in different positions for immune recognition. Although the mucin-like domain may be flexible, the equatorial rather than upward projection makes the mucin-like domain of MARV consistent with the attachment points of GP1 and GP2. These structural differences may explain why none of the current MARV vaccines can induce effective neutralizing antibodies. Gai et al. prepared virus-like particles assembled from GP and VP40 using the insect cell expression system [57]. VLP and *Poria cocos* polysaccharide (PCP-II) adjuvant was able to induce antibodies with pseudovirus neutralizing activity in rhesus monkeys. Their study showed that VLP could be one of the candidate prophylactic vaccines to prevent MARV infection. Zhu et al. generated recombinant PHV01 virus by replacing VSV GP with Musoke GP by reverse genetics [58]. Inoculation of guinea pigs with high (2 × 10^6^ PFU) or medium doses (2 × 10^4^ PFU) provided 100% and 97% protection, respectively. Their data suggest that immunization with PHV01 elicits a potent and dose-dependent neutralizing antibody response. Saito et al. tested the recombinant virus based on the VSV vector in Syrian hamsters to establish an alternative animal model that can be used to screen MARV vaccines [59]. The parental VSV caused severe and fatal disease in hamsters, and the LD_50_ value of parental VSV (10^2.8^ PFU) was lower than that of rVSV/MARV. Two of three animals infected with 10^6.5^ and 10^7.5^ PFU of rVSV/MARV succumbed within 4 dpi. Moreover, it was found that rVSV/MARV can cause severe hepatocyte necrosis as well as being able to replicate and proliferate in the liver, spleen, lungs, and brain. In both studies, the inoculation dose of PHV01 was much higher than that of rVSV/MARV, but they were suitable for different application scenarios. Given the safety concerns on live VSV recombinant virus vaccines above, we inactivated our vaccines in this study, although the three recombinant viruses were safe for 3-day-old suckling mice, and their immunogenicity is greatly reduced after inactivation.

To our knowledge, very few reports have isolated MARV-neutralizing antibodies and provided their sequences. Flyak et al. screened 19 MARV antibodies with neutralizing activity from B cells of human survivors, demonstrating that the Fab fragments of these antibodies all bound at or near the RBS [23]. This suggests that neutralizing antibodies recognize a single antigenic site and that their primary mechanism for neutralizing MARV may be inhibition of receptor-binding. In their study, 18 neutralizing antibodies could only weakly bind the GP of MARV. By contrast, the neutralizing antibody 60H07 elicited by rS-2A was screened among candidate antibodies with high-affinity. We wondered if this discrepancy was related to the different coatings in the binding assay, since Flyak et al. used GP, GPΔmuc, and GPpcl for binding reactions, while we used GP1.

The reason we chose GP1 was to obtain antibodies targeting the RBS site. This was inspired by a study by Hashiguchi et al., who demonstrated that the projection of the MARV mucin-like domain towards the equator makes the RBS on top of the GP more accessible than the EBOV [13]. We therefore believe that MARV is more likely to induce RBS-binding antibodies than EBOV. Combined with the published crystal structures of MARV and EBOV GPs, we believe that the recognition site of MARV-neutralizing antibodies generated by the human immune system must be near the RBS, but antibodies that recognize the RBS may not be able to neutralize MARV. Based on this speculation, we considered expressing the RBS of MARV, but the GP is a highly glycosylated protein, so that only expressing the RBS region may cause a significant difference in spatial structure. To overcome this, we designed the GP1 protein, which is characterized by the absence of a mucin-like domain and a GP2 subunit, but contains a glycan cap structure on GP1. Given that 60H07 can bind GP with higher affinity than the antibody obtained by Flyak et al., we speculate that the presence of the GP1 glycan cap may enhance the binding of the antibody to the RBS. Notably, there is also hope that this property may enhance the therapeutic effect when using a neutralizing antibody to treat MARV infection. Nevertheless, these issues and mechanisms need to be clarified and confirmed in future studies.

Although most vaccines have failed to induce MARV-neutralizing antibodies, these vaccines are still protective [60,61], demonstrating that the protective effect of vaccines is not limited to neutralizing antibodies. Keshwara et al. also RABV as the vector to construct FILORAB3, a bivalent vaccine carrying both RABV GP and MARV GP [60]. Without inducing neutralizing antibodies, FILORAB3 achieved 92% protection through antibody-dependent NK cell-mediated cytotoxicity (ADCC). Ilinykh et al. demonstrated that the human-derived non-neutralizing antibody MR228 fully protected mice from lethal challenge with MARV by mediating potent Fc effects, including ADNP (antibody-dependent neutrophil phagocytosis) and ADCP (antibody-dependent monocyte phagocytosis) [61]. These studies all point to the opsonization function of antibodies, which is mediated by Fc receptors on the surface of immune effector cells. However, most Fc receptors cannot bind to free antibodies. To obtain an activation signal, Fc receptors on effector cells need to bind to antibodies that have formed immune complexes, allowing Fc receptors to cross-link on the cell membrane, thereby triggering cellular responses. Therefore, the protective mechanism, including ADCC, requires many B cells expressing antigen-specific BCR to induce the killing effect of effector cells such as NK cells. We preliminarily showed that rS-MARV can generate large numbers of MARV GP-specific plasma cells and memory B cells, which have the potential to induce antibody opsonization. Therefore, rS-MARV may also have other protective effects, including those mediated by ADCC, ADNP, and ADCP.

Our results demonstrate that the vaccine encoding two copies of MARV GP effectively induced neutralizing antibodies in mice, indicating that rS-MARV is a potential candidate for a prophylactic vaccine. Ideal vaccines need to have a highly favourable risk-benefit for all age groups. Therefore, we preliminarily assessed the lethality of rS-MARV in mammary mice. Although 100% of 3-day-old suckling mice survived, we inactivated rS-MARV in consideration of biosafety and possible reversion to virulence. This makes rS-MARV potentially useful for pregnant women or malnourished immunosuppressed populations, which is an important consideration in MARV-endemic countries. We constructed the rVSV-Angola recombinant virus by replacing VSV's GP with Angola GP using the VSV reverse genetic system concerning the Takeshi Saito et al. study [59]. rVSV-Angola and golden hamsters were used together as an alternative lethal animal model to wild-type MARV to evaluate the protective effect of rS-MARV further. Golden hamsters were immunized with 50 μg of inactivated three rS-MARV recombinant viruses mixed with equal amounts of AddVax adjuvant. All animals received second and third booster immunizations two and four weeks after the initial immunization. Golden hamsters were injected intraperitoneally with 10^6^ TCID_50_ rVSV-Angola 3 weeks after the end of the final immunized. The three rS-MARVs showed 100% protection and an increasing trend in body weight in the animal model. The control animals died within 3 days after infection, with survival and body weight changes shown in Supplementary Figure 3A,B.

At the same time, we were the first to obtain 8 high-affinity MARV antibodies, including one neutralizing antibody, from fully antibody-humanized transgenic mice immunized with rS-2A. The predicted spatial structure of the neutralizing antibody indicates that it can successfully bind to the MARV GP of Ravn strain. The results of this study can therefore be used to guide new therapeutic and structure-based vaccine designs against MARV. Finally, these fully humanized antibodies may have clinical application potential in preventing MARV infection or treating MVD.

## Supplementary Material

Supplemental Material
