# Supplementary figures and images for: A rabies virus-vectored vaccine expressing two copies of the Marburg virus glycoprotein gene induced neutralizing antibodies against Marburg virus in humanized mice
Source: Emerg Microbes Infect. 2022 Dec 28;12(1):2149351. doi: 10.1080/22221751.2022.2149351 (PMC9809360; doi:10.1080/22221751.2022.2149351)

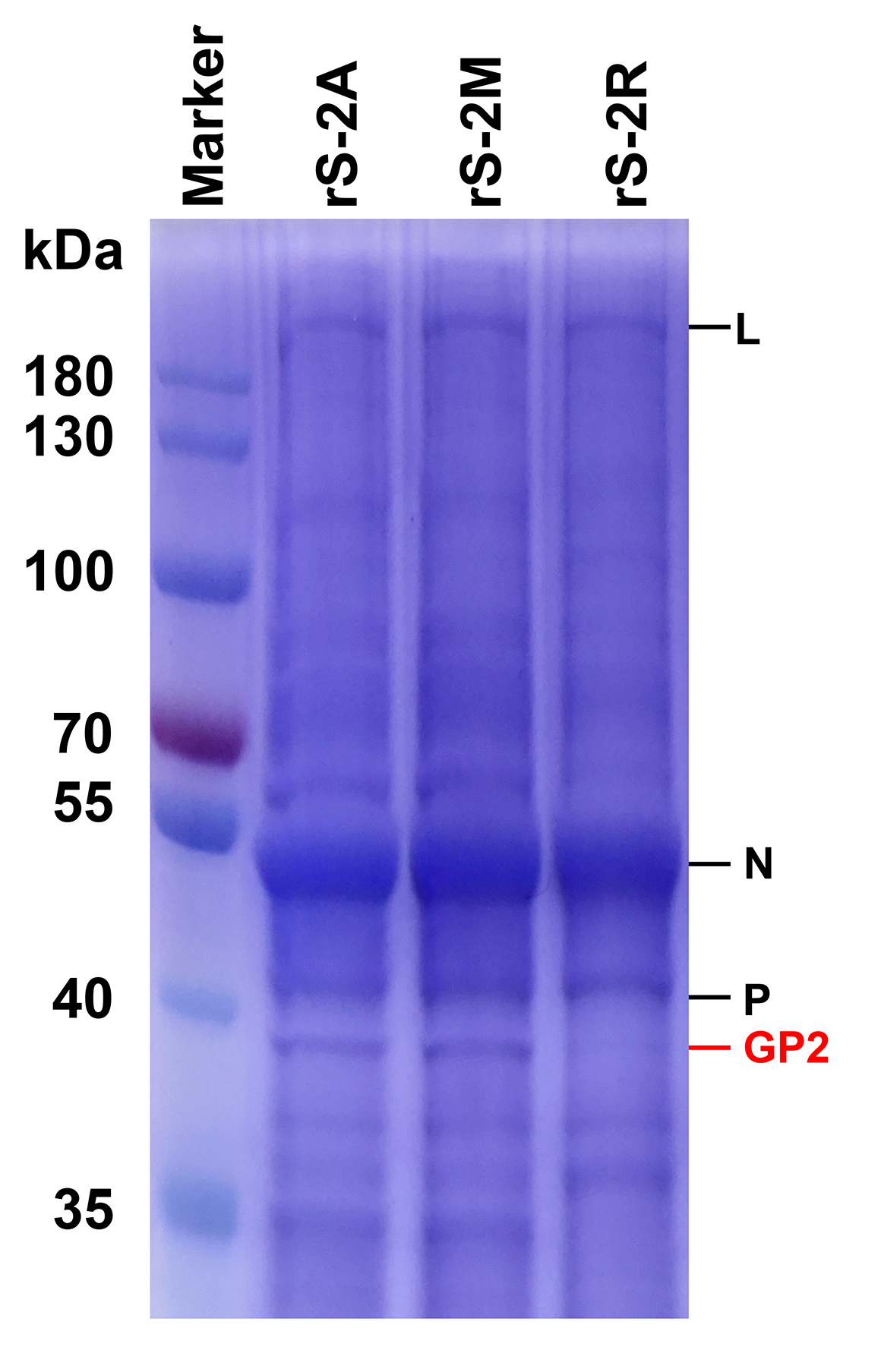

Supplement: Supplemental Material [file TEMI_A_2149351_SM6100.zip › Supplementary FIGURE 1.png]

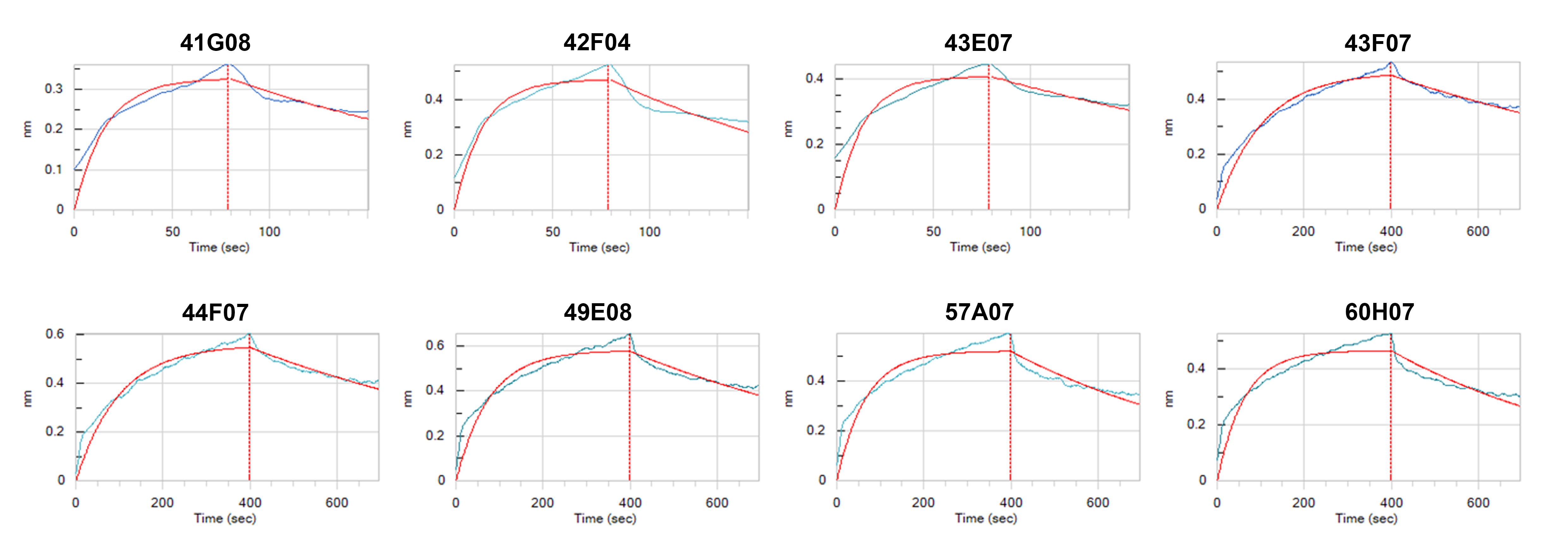

Supplement: Supplemental Material [file TEMI_A_2149351_SM6100.zip › Supplementary FIGURE 2.jpg]

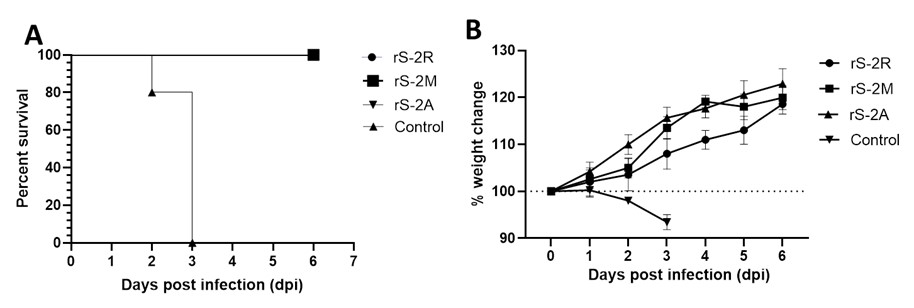

Supplement: Supplemental Material [file TEMI_A_2149351_SM6100.zip › Supplementary FIGURE3.jpg]
